# Determinants of postnatal care utilization in Ethiopia: a multilevel analysis

**DOI:** 10.1186/s12884-020-03254-7

**Published:** 2020-09-21

**Authors:** Gizachew Tadele Tiruneh, Alemayehu Worku, Yemane Berhane, Wuleta Betemariam, Meaza Demissie

**Affiliations:** 1 The Last Ten Kilometers (L10K) Project, JSI Research & Training Institute, Inc, Addis Ababa, Ethiopia; 2 Addis Ababa University School of Public Health, Addis Ababa, Ethiopia; 3 Addis Continental Institute of Public Health, Addis Ababa, Ethiopia

**Keywords:** Determinants, Ethiopia, Maternal health service, Postnatal care, Use of health service

## Abstract

**Background:**

The expansion of primary health care services in Ethiopia made basic health services available and accessible. The Last Ten Kilometers (L10K) project has strengthened the primary health care system through implementing innovative strategies to engage local communities to improve maternal and newborn health care behavior and practices in Amhara, Oromia, Southern Nations, Nationalities and Peoples [SNNP], and Tigray regions over a decade. Despite the efforts of the government and its partners to improve the use of maternal health services, the coverage of postnatal care is persistently low in the country. This study examined the individual and community level determinants for the persistently low uptake of postnatal care in the project areas.

**Methods:**

The study used a cross-sectional population-based survey that measured maternal and newborn health care practices among women who had live births in the last 12 months preceding the survey in Amhara, Oromia, SNNP, and Tigray regions. Multilevel random effects binary logistic regression analysis was used to assess the independent effects of community-and individual-level factors and moderating effects on the uptake of postnatal care.

**Results:**

This study identified region of residence, obstetric factors, and health service-related factors to be significant determinants for use of postnatal care. Obstetric factors include knowledge of obstetric danger signs (AOR: 1.30; 95% CI: 1.05–1.60), cesarean section mode of delivery (AOR: 1.96; 95% CI: 1.28–3.00), and institutional delivery (AOR: 10.29; 95% CI: 7.57–13.98). While the health service-related factors include attended family conversation during pregnancy (AOR: 1.48; 95% CI: 1.04–2.12), birth notification (AOR: 2.66; 95% CI: 2.15–3.29), home visits by community health workers (AOR: 1.98; 95% CI: 1.58–2.50), and being recognized as a model family (AOR: 1.27; 95% CI: 1.03–1.57).

**Conclusion:**

This study demonstrated that community-level interactions and promotive health services including antepartum home visits by community health workers, family conversation, birth notification, and model family, are important determinants to seek postnatal care. The findings also highlight the need for expansion of health facilities or design appropriate strategies to reach the disadvantaged communities. Program managers are recommended to strengthen community-based interventions to improve postnatal care utilization.

## Background

Most maternal and newborn deaths are largely preventable or treatable if skilled health care is provided during the intrapartum and early postnatal periods [1]. During the immediate postpartum period, critical interventions to end preventable maternal and newborn deaths need to be delivered [2]. However, in most developing countries, postnatal care (PNC) is the least prioritized program component of maternal and child survival interventions which is illustrated by a high rate of underutilization and discontinuity of maternal and child health programs [3]. Likewise, postnatal care has been poorly implemented in Ethiopia [4]. Despite the efforts of the government and its partners to improve the use of maternal health services through expansion of primary health care, except user free of maternal health services, strengthening the community health system to provide postpartum home visits, only 17% of women received PNC during the critical period of the first 2 days after delivery; and only 1% was visited by health extension workers, frontline community health workers. Less than 2% who delivered outside of the facility and 42% who delivered at health facility received PNC within 2 days. To make matters worse, of those who delivered in the facility, most did not receive proper PNC. More than two-thirds (69%) of women who had vaginal birth at the facility were discharged within 24-h after birth and commonly discharged within 6 h of delivery [5].

Evidence shows that the use of maternal and newborn health services during the postpartum period is influenced by many of socio-demographic, health service-related, and cultural factors [6–9]. Multiple studies find parity, mother’s education, and a woman’s economic status are significant predictors for the utilization of maternity care [10, 11]. Researchers also indicate that PNC utilization is affected by distance to facilities, area of residence, place and type of delivery [12–14], experience of problems during delivery, awareness about obstetric related danger signs, and awareness about PNC services [7, 9, 11]. Likewise, the use of antennal care (ANC), skilled attendance during childbirth, quality of ANC, and awareness of danger signs during ANC and delivery are found to be associated with seeking PNC service [15, 16].

Cultural beliefs regarding maternal health and illness are also other factors that can prevent women from utilizing modern maternal health care. The traditional postnatal confinement, and certain community rituals that take place during this period, hinder mothers from going to health facilities for PNC service [17].

Compared to antenatal care and skilled attendance at birth, research on postnatal care is limited in developing countries [18]. Moreover, most studies have largely ignored the potential effects of community-level factors that do not show a complete picture of the evaluation of the determinants affecting PNC utilization [19]. As such, this study intends to examine determining factors at individual and community-levels of the persistently low utilization of postnatal health services in Ethiopia.

## Methods

### Context

Ethiopia has expanded primary health care services through expansion of the Health Extension Program (HEP) and expansion of health centers and promotion of early healthcare-seeking through community mobilization to reach most communities and households and provide preventive, promotive, and basic curative services. The promise of the HEP has been the graduation of model families where health extension workers (HEWs) train households to acquire the necessary knowledge, skills, and behavior change in health practices. When these households demonstrate practical changes in the use of health service programs: environmental health, personal hygiene, and serve as models in their community, then they graduate. To reach more communities, this strategy has been accelerated through the participatory engagement of model families that are early adopters of desirable health practices and have acceptance and credibility by their community and the women development army (WDA) group. The WDA network builds on the critical mass of model families and creates volunteer leaders that scale-up the dissemination of knowledge, innovation, and service utilization through social networks. They are actively engaged in promotion and prevention activities as well as social mobilization efforts to expand HEP deeper into communities and families and ensure community ownership [20].

The Last Ten Kilometers (L10K) project has been supporting in strengthening the HEP through the implementation of innovative strategies to engage local communities to improve high-impact reproductive, maternal, newborn and child health (RMNCH) care behavior and practices in 115 *woredas* (i.e., districts) in four of the most populous regions of Ethiopia (i.e., Amhara, Oromia, Southern Nations, Nationalities and Peoples [SNNP], and Tigray) since 2008. During the first funding cycle, i.e., between 2008 and 2015, the Project implemented innovative strategies to engage local communities to reach its objectives. Three community-based strategies—Community-Based Data for Decision-Making (CBDDM), family conversation, and birth notification—were implemented in the 115 woredas covering about 3070 *kebeles,* the lowest administrative unit.

Community-based data for decision-making, introduced in July 2013, was used to identify pregnant women and to ensure they received antenatal, intrapartum, and postpartum care [21]. CBDDM fostered the kebeles to generate and use data to improve maternal and newborn health practices. The strategy identified underserved households and linked them with HEWs and kebele managers helping to address barriers in accessing maternal and newborn health services. Accordingly, HEWs were trained to support WDA team leaders to map 30 households in their catchment areas, to keep each household under surveillance, and to ensure the provision of maternal and newborn health services along the continuum of care. The surveillance system used images so that they could be maintained and updated by individuals with little or no education. HEWs collect data from WDA team leaders’ surveillance to help kebele leaders to identify and address barriers that make access to maternal and newborn health services difficult.

The CBDDM strategy has resulted in improvements in institutional deliveries and newborn health care behaviors and practices. However, there was no evidence of any effect of the intervention on postnatal care within 2 days of childbirth [21]. Accordingly, alternative strategies, complementary to CBDDM, such as family conversation and birth notification, were designed to promote maternal and newborn health behaviors. Family conversation, on the other hand, is a forum conducted at the house of a pregnant woman with her family members and relatives who are expected to support her during her pregnancy, labor, delivery, and postpartum period. Family conversation session was introduced early in 2014 to promote birth preparedness and the use of early postpartum care and essential newborn care. Birth notification strategy was introduced by mid-2014 to promote early postnatal care. Since October 2015, L10K focused on institutionalizing the CBDDM strategy and strengthen the implementation of family conversations and birth notification strategies in the 115 woredas [21]. During the intervention period, L10K Project’s contribution has shown an increase in skilled delivery from 10 to 67% between 2011 and 2017. Nevertheless, despite the efforts and huge investments made to improve the use of maternal health services, only one-third of mothers received PNC within 48 h [22].

### Design

This study used a cross-sectional population-based design. The study population consists of women who had live births in the last 12 months preceding the survey.

### Sample size and sampling

We used the data that was collected from the representative sample of 2724 women aged 15 to 49 years, from 298 Kebeles. The data were drawn from a cross-sectional household survey datasets representing the 115 woredas (where L10K operates) and were collected from October–November 2017. The survey employed a two-stage stratified cluster sampling method. The details of the design are described somewhere else [23].

The adequacy of the sample size, required to address this study’s objective, was checked using StatCalc for a double population formula for comparative cross-sectional study design considering different exposure variables including birth order, place of delivery, maternal education, and wealth quintile from the 2016 EDHS report [5]. The assumption made to calculate sample size was considering a 95% confidence level (Zα/2 = 1.96), and design effect (D = 2), power of 80%. Based on Anderson’s health-seeking behavior model [24], the researchers assumed first birth order, home delivery, no maternal education, and poorest wealth quintile as exposure and highest birth order/6+, facility delivery, higher education, and richest wealth quintile as non-exposure. Adding a 10% non-response rate, the maximum sample size obtained by birth order was 750. Accordingly, we ensured that the available sample was adequate to address this objective.

### Data collection

Data were collected through interviews of eligible mothers aged 15–49 years. During the interview, information about household and socio-demographic characteristics of mothers, awareness, and experiences related to the women’s use of maternal health services, was collected from women with children in their first year of life. The data were collected using a structured interview questionnaire (Additional file 1) and archived using a web-based mHealth platform (i.e., SurveyCTO) using smartphones.

### Measurement

The outcome variable of interest was, the proportion of women and their newborns who received postpartum care at the health facility or their home within 6 weeks of delivery. It is measured interview of women weather pre-discharge care provided for them and their newborns after 24-h of stay for whom delivered at the health facility and any postnatal check-up for mother’s and newborn’s health by a health care provider within 6 weeks of giving birth.

The independent variables considered include individual and community level factors. The individual socio-demographic variables include maternal education, husband’s education, religion, marital status, household wealth index, and distance to health facility. Obstetric characteristics include mode of delivery, place of delivery, utilization of ANC services, knowledge of obstetric danger signs, and encountered obstetric complications (during pregnancy, childbirth, or postpartum), as well as program characteristics including community health workers (CHWs) home visit, model family, and birth notification, were considered. Administrative region and area of residence (clusters/kebeles) were considered as community-level random components.

Model families are defined as those households who received training from HEWs, acquire the necessary knowledge, skills, and behavior in health practices and demonstrate practical changes in the use of health service programs and serve as models in their community. Family conversation is a forum conducted at the house of a pregnant woman with her family members and relatives who are expected to support her during pregnancy, labor, delivery, and the postpartum period. It creates an opportunity to discuss issues such as birth preparedness and essential newborn practices with all these family members together. It is measured by asking women regarding their attendance of at least one family conversations at home during their last pregnancy. While birth notification is a strategy introduced to promote early postnatal care. It is measured through interviews of women weather they took measures to inform the HEW about their childbirth immediately after delivery or not.

A wealth index score was constructed for each household with the principal component analysis of the household’s possessions (electricity, watch, radio, television, mobile phone, telephone, refrigerator, table, chair, bed, electric stove, and kerosene lamp), and household characteristics (type of latrine, water source, floor, and wall material). Subsequently, households were ranked according to wealth score and then divided into five quintiles using Principal Component Analysis (PCA) method.

### Data analysis

#### Individual-level analysis

Data were analyzed for both descriptive and inferential statistics using Stata version 15.1. Socio-demographic data were summarized by frequency tables and summary statistics. The researcher conducted an individual-level analysis to assess the association between the individual and contextual characteristics of the respondents with PNC use using Pearson’s Chi-squared statistics taking the complex survey nature into account.

Post-stratification sampling weights were used to adjust the non-proportional allocation of the sample to the different regions and weighted analysis was used to ensure the representativeness of the survey estimates.

#### Model specification and assumptions

Multilevel bivariate and multivariable analysis using cluster-level random effects logistic regression models were used to assess the independent effects of community factors and moderating effects on the association between individual variables and PNC. The models were adjusted for survey design and individual and contextual characteristics of the respondents. To assess the contribution of the respondents’ characteristics to the model, a two-level logistic regression model was applied (i.e., individuals (level 1) were nested within communities (level 2).

In fitting models, iterative based backward elimination and forward selection methods were used to select explanatory variables. For the purpose, the independent variables were retained in each of the models if the *p*-value was less than 0.2. Accordingly, maternal education, household wealth index, distance to health facility, family currently recognized as model, family conversations during pregnancy, knew at least 3 obstetric danger signs, encountered obstetric complications, CHW (either by HEWs or WDAs) home visit during pregnancy, ANC visit, place of delivery, mode of delivery, and birth notification, were retained in the model.

#### Multilevel analysis

Both random-intercept and random coefficient logistic models were fitted to estimate associations between the individual and community variables and the likelihood of receiving PNC using *xtlogit and xtmelogit* Stata commands. The null model is fitted without the explanatory variable. The random-intercept logistic models were fitted to assess the influence of unobserved community-level characteristics on the overall variation in PNC utilization allowing the probability of receiving PNC to vary randomly across communities assuming the effects of individual characteristics are the same in each community, i.e. the coefficients of all explanatory variables are fixed across communities. While the random coefficient model was fitted for household wealth (mean-centered), allowing to vary across communities. Finally, we adjusted both individual and community variables and a cross-level interaction between region and distance to the health facility to see any evidence of effect modification of the association between distance to health facility and PNC utilization by region.

The likelihood ratio test showed that the effect of wealth index variables significantly varied across communities indicating the random coefficient model provides a better fit than the random intercept model (*p* = .005). Moreover, the model fit statistics, AIC, reduced as compared to intercept models indicating that allowing wealth index to vary across communities increased the predictive ability of the multilevel models for PNC. Moreover, we noticed that the intercept models do not provide additional information. As such, we reported random coefficient models.

Measures of associations were expressed as odds ratio (OR) and 95% confidence interval (CI). Important assumptions and fitness of such statistical models were checked using the standard procedures. The goodness-of-fit of the models was assessed using the global Wald’s statistics, the likelihood ratio test of the cluster-level random effects, and sensitivity of the quadrature approximation that was used to estimate the models.

## Results

### Sociodemographic characteristics

A total of 2724 women with live births in the 12 months preceding the survey were interviewed. Most of the respondents (82.9%) were aged 34 years or below. The majority did not have any education (58%) and about half of them lived within 30 min walking distance from a health post or health center (49.1%). The majority of the respondents were Orthodox Christians (59.6%), followed by Muslims (20.9%), and then Protestant Christians (18.8%). (Table 1).

**Table 1:** Background characteristics of women who had live birth in the 12 months preceding the survey in Ethiopia, 2017

Background characteristics	Number of women (n)	Percentage (%)
*Maternal age*		
< 20	161	5.9
20–34	2098	77.0
35–49	465	17.1
*Education*		
No education	1571	57.7
Primary	595	21.8
Higher	558	20.5
*Religion*		
Orthodox	1623	59.6
Protestant	512	18.8
Muslims	568	20.9
Traditional/others	21	0.8
*Wealth quintile*		
Poorest	545	20.0
Poorer	546	20.0
Middle	544	20.0
Richer	551	20.2
Richest	538	19.7
*Walking distance to nearest health facility (health post or health center)*		
< 30 min	1337	49.1
> =30 min	1387	50.9
*Region*		
Amhara	929	34.1
Oromia	713	26.2
SNNP	687	25.2
Tigray	395	14.5
**Total**	**2724**	**100.0**

### Obstetrics characteristics

More than three-quarters of the women (77.1%) had two or more children. Most (91.2%) women attended ANC at least once during pregnancy, while about 52% continue to have at least four ANC consultations. A little less than two-thirds of mothers (66.1%) delivered at health facility and about 5.6% of these delivered through cesarean sections. A little more than half of the mothers (55.1%) mentioned at least three obstetric danger signs during pregnancy, childbirth, or postpartum. About 8.4% of women encountered any obstetric complications during pregnancy, childbirth, or postpartum (Table 2).

**Table 2:** Distribution of women who had live birth within the last 12 months preceding the survey in Ethiopia, by obstetric characteristics, 2017

Obstetric characteristics	Number of women (n)	Percentage (%)
*Number of children*		
1	624	22.9
2–3	877	32.2
4+	1223	44.9
*Mentioned at least 3 pregnancy danger signs*		
Yes	883	32.4
No	1841	67.6
*Mentioned at least 3 childbirth danger signs*		
Yes	1062	39.0
No	1662	61.0
*Mentioned at least 3 postpartum danger signs*		
Yes	1180	43.3
No	1544	56.7
*Mentioned at least 3 pregnancy, intrapartum, or postpartum danger signs*		
Yes	1501	55.1
No	1223	44.9
*Encountered complications during pregnancy*		
Yes	208	7.7
No	2515	92.3
*Encountered complications during childbirth*		
Yes	153	5.6
No	2570	94.4
*Encountered complications after childbirth*		
Yes	107	3.9
No	2616	96.1
*Encountered complications during pregnancy, childbirth, or after childbirth*		
Yes	228	8.4
No	2495	91.6
*Mode of delivery*		
Caesarean section delivery	152	5.6
Vaginal delivery	2572	94.4
**Total**	**2724**	**100.0**

### Health-service related factors

A little less than one-third (31.8%) of families were model families at the time of the survey; less than 10% of mothers had at least one family conversation during pregnancy. Likewise, about 41.1% of births were notified to HEWs immediately after the mother delivered, either by the mother herself or anyone from her family or neighbor, for early PNC. Only a little higher than one-third (36.9%) of women received home visits during pregnancy by CHWs (Table 3).

**Table 3:** Percent distribution of women who had live birth within the last 12 months preceding the survey in Ethiopia, by health-service related factors, 2017

Health-service related factors	Number of women (n)	Percentage (%)
*Antenatal care utilization (at least one)*		
At least one	2481	91.2
None	240	8.8
*Antenatal care utilization (4+)*		
At least 4 visits	1387	52.3
Less than 4 visits or none	1266	47.7
*Place of delivery*		
Facility	1791	66.1
Home or elsewhere	919	33.9
*CHW (HEWs or WDAs) home visit during pregnancy*		
Yes	1006	36.9
No	1718	63.1
*Used motorized transport for delivery*		
Yes	1111	57.0
No	838	43.0
*Family currently recognized as model family*		
Yes	867	31.8
No	1857	68.2
*Family conversations during pregnancy*		
Yes (at least 1)	240	8.8
No	2283	91.2
*Birth notification*		
Yes	1119	41.1
No	1604	58.9
**Total**	**2724**	**100.0**

### Postnatal care service utilization

Overall, about 36.8% (95% CI: 35.0–38.6) of women received PNC service within 6 weeks after delivery—29.4% (95% CI: 27.7–31.1) of these received the service within 48 h and 34.1% (95% CI: 32.3–35.8) within 7 days. About one-third (34%) of the mothers received one PNC visit; only 2 and 1% received two and three PNC visits, respectively. Most of the mothers received PNC service at health facilities; 29% at the health facility and only 7% received at home. About a quarter (27%) of mothers received PNC after birth in a health facility staying for at least 24-h.

### Factors associated with postnatal care utilization

As presented in Table 4 below, Model 1, the intra-class correlation coefficient (ICC) showed that the community variance in PNC was estimated at 22%. It means that 22% of the residual variation in the propensity to use PNC services is attributable to unobserved community characteristics. The likelihood ratio test showed that there is strong evidence that the between community variance is non-zero (chi2 = 202.78; *p* < .01).

**Table 4:** Multilevel logistic regression results on the predictors of postnatal care in Ethiopia, 2017

Characteristics	Model 1 Null model	Model 2 Coefficient model	Model 3 Interaction effects
*Maternal education*			
No education		1.00	1.00
Primary		0.95 (0.74–1.21)	0.94 (0.73–1.21)
Higher		0.97 (0.75–1.26)	0.97 (0.74–1.26)
*Wealth quintile*			
Poorest		1.00	1.00
Poorer		0.88 (0.63–1.23)	0.87 (0.62–1.22)
Middle		0.83 (0.59–1.216	0.81 (0.57–1.14)
Richer		0.96 (0.67–1.37)	0.94 (0.65–1.35)
Richest		0.73 (0.51–1.06)	0.71 (0.49–1.03)
*Distance to nearest health facility*			
< 30 min		*1.24 (1.01–1.52)*	*0.84 (0.55–1.28)*
> =30 min		1.00	1.00
*Family recognized as model family*			
Yes		*1.27 (1.03–1.57)*	*1.27 (1.03–1.57)*
No		1.00	1.00
*CHW home visit during pregnancy*			
Yes		*1.98 (1.58–2.50)*	*1.98 (1.57–2.50)*
No		1.00	1.00
*At least one family conversations happened at home during pregnancy*			
Yes		*1.48 (1.04–2.12)*	*1.49 (1.04–2.13)*
No		1.00	1.00
Mentioned at least 3 antepartum, intrapartum, or postpartum danger signs			
Yes		1.30 (1.05–1.60)	1.31 (1.06–1.61)
No		1.00	1.00
*Encountered complications during pregnancy, childbirth, or after delivery*			
Yes		*1.23 (0.93–1.62)*	*1.21 (0.92–1.61)*
No		*1.00*	*1.00*
*Birth notification*			
Yes		*2.66 (2.15–3.29)*	*2.69 (2.17–3.34)*
No		1.00	1.00
*Antenatal care utilization*			
At least 4 visits		*1.04 (0.84–1.28)*	*1.03 (0.83–1.28)*
Less than 4 visits or none		1.00	1.00
*Place of delivery*			
Facility		*10.29 (7.57–13.98)*	*10.47 (7.69–14.26)*
Home or elsewhere		1.00	1.00
*Mode of delivery*			
Vaginal delivery		1.00	1.00
Cesarean section delivery		*1.96 (1.28–3.00)*	*2.00 (1.30–3.05)*
*Community characteristics*			
*Region*			
Amhara		1.00	1.00
Oromia		2.99 (2.07–4.32)	2.51 (1.55–4.06)
SNNP		1.34 (0.93–1.94)	1.06 (0.66–1.72)
Tigray		1.30 (0.87–1.93)	0.85 (0.51–1.41)
Distance to health facility* Region			
< 30 min*Oromia			1.37 (0.77–2.45)
< 30 min*SNNP			1.53 (0.86–2.72)
< 30 min*Tigray			2.28 (1.25–4.14)
Community level intercepts (SE)	*0.66 (0.05)	*0.02 (0.01)	*0.03 (0.01)
*Random effects*			
Coefficient variance (SE)	**–**	*0.03 (0.02)	*0.03 (0.02)
Community level variance (SE)	*0.91 (0.14)	*0.46 (0.11)	*0.48 (0.11)
Covariance (SE)	**–**	*-0.12 (0.04)	*-0.13 (0.04)
Log-likelihood	**202.78**	**60.09**	**62.83**
*Model fit statistics*			
ICC (SE)	**0.22 (0.03)**	**0.12 (0.02)**	**0.13 (0.03)**
AIC	**3494.46**	**2761.47**	**2759.93**

Model 2 showed that PNC utilization was higher amongst mothers residing within 30 min walking distance from the nearest health facility (health post or health center) (AOR: 1.24; 95% CI: 1.01–1.52). Women who mentioned at least 3 antepartum, intrapartum or postpartum danger signs had 30% higher odds of receiving PNC (AOR: 1.30; 95% CI: 1.05–1.60). The odds of receiving PNC was 10 times higher among those delivered at health facility (AOR: 10.29; 95% CI: 7.57–13.98); more than 2 times higher among women whose birth was notified to HEWs (AOR: 2.66; 95% CI: 2.15–3.29); and 96% higher among women who delivered through cesarean section (AOR: 1.96; 95% CI: 1.28–3.00). Moreover, PNC utilization was higher amongst families recognized as model family (AOR: 1.27; 95% CI: 1.03–1.57) and those who had family conversation during pregnancy (AOR: 1.48; 95% CI: 1.04–2.12), had higher odds of using PNC than their counterparts. However, the association between maternal education, wealth quintile, and PNC were not statistically significant.

Women who live in Oromia were 2.99 times higher odds of using PNC than those mothers who live in Amhara region (AOR: 2.99; 95% CI: 2.07–4.32). However, the association between living in SNNP and Tigray region and PNC use were not statistically significant.

The cross-level interaction model, Model 3, revealed that there is evidence of effect modification of the association between distance to health facility and PNC utilization by region. Within the community effect of distance to the health facility, as well as within the community effect of the administrative region in Tigray disappears. In Tigray region, the odds of receiving PNC were significantly higher in those women who resided within 30 min walking distance to health facility as compared to women who resided further away from the facility (AOR: 2.28, 95% CI 1.25–4.14).

## Discussion

Region of residence, obstetric, and service-related factors are the major determinants for the use of PNC among rural women in Ethiopia.

The cross-interaction model showed that region modified the association between distance to facility and PNC. The association between proximity of women to health care facilities and uptake of PNC significantly varied across regions. Women who resided near facilities in Tigray region were significantly receiving PNC as compared to communities who live far from the facility. This suggests the existence of differential geographic access to PNC, despite the program being pro-poor, as documented in this study and other studies in Ethiopia [25]. Mothers in Tigray region have better access to health facility and receiving PNC or mothers who lived near a facility in Tigray might be motivated to visit health facilities and receive services or HEWs are motivated to visit them at home disproportionally. Previous studies in developing countries also suggest that where health services are brought closer to the people, it could improve the uptake of the services including PNC [19, 26]. As such, it underlines the need for expansion of health facilities or designs appropriate strategies to reach the disadvantaged communities.

Illness-level factors such as knowledge of obstetric danger signs and delivery through cesarean section are determining the use of PNC service. Mothers who delivered through cesarean section were more likely to receive PNC services than mothers who delivered through spontaneous vaginal delivery. As documented in this study, about 73% of the women who received PNC at a health facility, stayed there after delivery. Mothers likely received PNC before discharge, would be delivered by cesarean section. Previous studies in rural Ethiopia [12–14] also showed women who had cesarean births used more postpartum care than those who had vaginal births. The result of this study revealed that the use of PNC services has been significantly influenced by knowledge of antepartum, intrapartum, or postpartum danger signs This is in line with the studies carried out in Ethiopia [13, 14]. An integrative review by Adams and Smith suggested that awareness about pregnancy, childbirth, and/or postpartum complications is one of the common determinants of postpartum care use in developing countries [27].

The service-related factors—family conversation; CHWs’ home visit during pregnancy; and HEWs being notified of the birth, were predicting factors for the use of PNC. Participating in at least one family conversation during pregnancy was associated with the use of PNC services. The effect of family conversation on early PNC visits was also documented in earlier studies in Ethiopia [28, 29]. A similar study suggests that family conversations could potentially play a crucial role in changing a family’s behaviors towards better maternal and newborn care practices through dismantling family healthcare decision-making barriers discussing birth preparedness and essential newborn practices with family members together [28]. Another intervention study on community-based collaborative approach involving HEWs and community health development agents illustrates that having two to four community maternal and newborn health family meetings during pregnancy with a family member was associated with the receipt of PNC [29]. Likewise, mothers/relatives/WDAs who informed HEWs about the birth immediately after delivery were about 2 times more likely to use PNC than their counterparts. This is in agreement with a previous observational study of a collaborative community-based quality improvement intervention in Ethiopia which involved family meetings and labor and birth notification contributed to the increased receipt of PNC within 48 h by skilled providers or HEWs [30]. This notification system might motivate HEWs to do home visits for the provision of PNC to the mother and the newborn.

Being recognized as a model family was associated with the uptake of PNC. This shows the contribution of the HEP in mobilizing the community and HEWs efforts of educating families on uptake of PNC. Model families are early adopters of the HEP education and package of services as well as role models to their communities. Their knowledge, skills, and behavior change in health practices they gained and their models in their community would influence the uptake of PNC [20]. Previous studies in Ethiopia also demonstrate that model family was more likely to use the HEP [31] and maternal health services [32].

Community health workers’ home visits during pregnancy where they discussed health-related issues, were statistically significantly linked with the use of PNC. A study in Tanzania also showed that counseling by a CHW to go to a facility for PNC was associated with PNC utilization [33]. Other studies in Ethiopia demonstrate that women who receive information about PNC services, utilize PNC [25]. A randomized trial by McConnell et al. [34] in Kenya found that CHW-administered postnatal checklists lead to better recognition of postnatal problems and more likely to seek care.

Antenatal care visits and institutional delivery would have a positive impact on the utilization of postpartum healthcare services if adequate counseling was provided regarding the need to seek PNC and avoid possible postpartum complications [9, 19, 26]. The odds of receiving PNC is higher among women who delivered in health facility. This might be due to mothers were advised about the need to have a follow-up visit before being discharged from health facility after delivery or birth notification after delivery to HEWs to do PNC home visits as documented in this study. However, like other studies elsewhere [35], this study shows that antenatal consultations were not associated with uptake of PNC which might be because mothers were not likely informed about the importance of PNC; its availability; recommended timing; and targeted frequency of postnatal visits, during their antenatal visits and before being discharged from health facility after delivery. These were documented by previous studies [35, 36]. This suggests that not enough emphasis is being given to the importance of PNC during antepartum contact, which is a missed opportunity for the health system to increase uptake of PNC.

Common demographic characteristics including maternal age, maternal education, and household wealth did not determine the utilization of PNC. Though this finding is congruent with the national pro-poor health policies and strategies, there is no concluding evidence in the global literature that these characteristics are determining the use of PNC. Some studies in Ethiopia [14, 25] and elsewhere [33] showed that demographic characteristics like marital status, maternal age, education, ethnicity, income, prior obstetric history, and cultural practices, did not have a significant association with the utilization of PNC. A recent systematic review of studies in Ethiopia also showed that maternal education did not have a significant association with the use of PNC [9]. This might have related to the PNC service delivery strategy. One thing maternal health services in Ethiopia are provided free of charge and another thing is as most of the women receiving PNC associated with their delivery, pre-discharge care, and home-based PNC.

Applying advanced analytic techniques, multilevel cluster-level random effect models, we estimated the effects of contextual variables and their interactions with individual-level variables on the use of PNC in Ethiopia which is critical to developing effective strategies to improve the delivery of PNC in the country. Despite the efforts to minimize biases using memory aids, pretested the survey tools, trained data collectors, and allocated adequate days for the primary data collection, the findings of this study may have been affected by social desirability and recall biases.

## Conclusions

In conclusion, this study identified multiple factors- obstetric, health service-related, and region of residence that influence PNC utilization in rural Ethiopia. Efforts to improve PNC coverage must consider a comprehensive intervention package that promotes utilization of services at the community and facility levels.

Exploring women’s perspectives on PNC service in general and the acceptability and feasibility of keeping women and newborns for at least 24 h at health facilities for postpartum observation, in particular, would be helpful.

## Supplementary information

**Additional file 1:.** Survey Questionnaire.doc. Survey questionnaire we used to collect information from respondents.

## Data Availability

The raw data used for this study is available upon reasonable request from the corresponding author.

## Abbreviations

ANC: Antenatal care
CBDDM: Community-based data for decision making
CHW: Community health worker
CI: Confidence interval
EDHS: Ethiopian demographic and health survey
HEP: Health extension program
HEW: Health Extension Worker
ICC: Intra-class correlation coefficient
L10K: Last Ten Kilometers Project
OR: Odds ratio
PNC: Postnatal care
RMNCH: Reproductive, maternal, newborn and child health
SNNP: Southern Nations, Nationalities, and Peoples’
WDA: Women’s development army
