# Supplementary material for: Determinants of postnatal care utilization in Ethiopia: a multilevel analysis
Source: BMC Pregnancy Childbirth. 2020 Sep 21;20:549. doi: 10.1186/s12884-020-03254-7 (PMC7507276; doi:10.1186/s12884-020-03254-7)
Supplement: Supplementary file 1 — Additional file 1:. Survey Questionnaire.doc. Survey questionnaire we used to collect information from respondents. [file 12884_2020_3254_MOESM1_ESM.doc]

#### Household Survey Questionnaire

| Question | Answer |
| --- | --- |
| IDENTIFICATION and CONSENT |  |
| Name of the household head |  |
| Region? | |  | Amhara | Amhara | | --- | --- | --- | |  | Oromia | Oromia | |  | SNNP | SNNP | |  | Tigray | Tigray | |
| Date of data collection |  |
| Do you agree to participate? | 1. Yes 2. No |
| q104_w0_11. What is the name of your infant (0 to 11 months)? |  |
| q105_w0_11. Sex of [q104_w0_11] | |  | 1 | Male 2. Female | | --- | --- | --- | |
| q106_w0_11. What is the age of [q104_w0_11]?  Remember to answer in MONTHS. Between 0 to 11 months; enter '0' if age of the child is less than one month  Response constrained to: . <12 and . >=0 |  |
| q108_w0_11. Verify child’s date of birth by asking to see the child’s health/immunization card | |  | 1 | Child’s date of birth verified | | --- | --- | --- | |  | 2 | Not possible to verify | |
| Section 2: Background and Household Characteristics |  |
| q202. How old were you on your last birthday?  Between 15 and 49 (years)  Response constrained to: . < 50 and . > 14 |  |
| q203. How long (in years) have you been living continuously in (NAME OF CURRENT PLACE OF RESIDENCE)?  If the respondent has always lived here, enter 95. If the respondent has been living here for less than one year, enter 0.  Response constrained to: .=95 or . =0 or .< ${q202} |  |
| q204. Are you able to read or write a simple sentence? | |  | 1 | Yes 2. No | | --- | --- | --- | |
| q205. Did you ever attend normal school?  Question relevant when: ${q204} =1 | | 1 | Yes 2. No | | --- | --- | |
| q206. What is the highest grade you completed?  Question relevant when: ${q205} =1 |  |
| q207. What is your religion? | |  | 1 | Orthodox | | --- | --- | --- | |  | 2 | Catholic | |  | 3 | Protestant | |  | 4 | Muslim | |  | 5 | Traditional | |  | 6 | Other | |
| q208. Are you currently married or living together with a man as if married? | |  | 1 | Yes, currently married | | --- | --- | --- | |  | 2 | Yes, living with a man | |  | 3 | No, not in union | |
| q209. Is your husband living with you now or is he staying elsewhere?  Question relevant when: ${q208} =1 | |  | 1 | Living together | | --- | --- | --- | |  | 2 | Staying elsewhere | |
| q205h. Did your husband ever attend formal school?  Question relevant when: ${q208} =1 | |  | 1 | Yes | | --- | --- | --- | |  | 2 | No | |
| q206h. What is the highest grade your husband completed?  -  Question relevant when: ${q205h} =1 |  |
| q210. How old were you when you first married?  enter 11 if less than 11 years; age of marriage cannot be higher than the age of the women  Question relevant when: ${q208} =1  Response constrained to: . >10 and .< ${q202} or .= ${q202} |  |
| q210a. How old was your husband when you were first married?  Add 99 if Do not know  Question relevant when: ${q208} =1  Response constrained to: .>12 and .<80 or .=99 |  |
| q211. How many times were you pregnant?  Including those that did not end with a live births; record 14 if more than 14 times  Response constrained to: .>0 and .<15 |  |
| q212. How many times have you given live birth?  I mean, to a child who ever breathed or cried or showed other signs of life – even if he or she lived only a few minutes or hours]  Response constrained to: .< ${q211} or .= ${q211} |  |
| q213. How old were you when you first gave a live birth?  enter 10 if less than 10 years  Response constrained to: .< ${q202} or .= ${q202} and .>9 |  |
| q214. Have you ever given to a live birth last year (other than [q104_w0_11]?  I mean, to a child who ever breathed or cried or showed other signs of life – even if he or she lived only a few minutes or hours | 1. Yes 2. No |
| q215. Is the child born last year alive?  Question relevant when: ${q214} =1 | 1. Yes 2. No |
| q216d_qualify. Did the child die before a month old?  Question relevant when: ${q215} =2 | 1. Yes 2. No |
| q216d. At how many days did the child die  Enter 0 if died before 1 day  Question relevant when: ${q216d_qualify} =1  Response constrained to: .<31 |  |
| q216m. At how many months did the child die?  Between 1 and 11 months  Question relevant when: ${q216d_qualify} =2  Response constrained to: .<12 |  |
| Have you ever given stillbirth?  I mean, to a child who never breathed or never cried or showed any signs of life | 1. Yes 2. No 8. Don’t know |
| Have you ever given stillbirth last year?  Question relevant when: ${everstillbirth} =1 | 1. Yes 2. No |
| q217m. Total number of boys ever born?  Includes still births; cannot exceed the number reported in q211 |  |
| q217f. Total number of girls ever born?  Includes still births; sum of boys and girls cannot exceed the number reported in q211  Question relevant when: ( ${q211} - ${q217m} )>0  Response constrained to: .<=( ${q211} - ${q217m} ) |  |
| q217hhtot. How many people are in the household?  Persons who usually live in the household  Response constrained to: .<15 and .>0 |  |
| q217chlsch. Do all children ages 6 to 12 attend school? | |  | 1 | Yes 2. No | | --- | --- | --- | |  | 3 | No children ages 6 to 12 | |
| q218. What is the main source of drinking water for members of your household?  - | |  | 1 | Piped into dwelling | | --- | --- | --- | |  | 2 | Piped into compound | |  | 3 | Piped outside compound | |  | 4 | Covered Well | |  | 5 | Protected Spring | |  | 6 | Protected hand pump spring | |  | 7 | Open Well | |  | 8 | Open Spring | |  | 9 | River | |  | 10 | Pond/Lake/Dam | |  | 11 | Rainwater | |  | 12 | Other | |
| q218oth. Specify other water source  Question relevant when: ${q218} =12 |  |
| q219. Is the source of water available in the compound?  Question relevant when: ${q218} !=1 and ${q218} !=2 | |  | 98 | Yes, water source in the compound | | --- | --- | --- | |  | 2 | No | |
| q219h. How many hours does it take to get water?  enter '0' if less than one hour'; round to the nearest hour; enter 6 if more than 6 hours  Question relevant when: ${q219_comp} =2  Response constrained to: .<7 |  |
| q219m. How many minutes does it take to get water?  Question relevant when: ${q219h} =0  Response constrained to: .<61 and .>0 |  |
| q220. Do you treat your water in any way to make it safer to drink? | |  | 1 | Yes 2. No | | --- | --- | --- | |
| q221. What do you usually do to the water to make is safer to drink at home?  -  Question relevant when: ${q220} =1 | |  | 1 | Boil | | --- | --- | --- | |  | 2 | Add bleach/chlorine | |  | 3 | Strain it through a cloth | |  | 4 | Use water filter (ceramic, sand, composite, etc.) | |  | 5 | Solar disinfection | |  | 6 | Let it stand and settle | |  | 7 | Other | |  | 8 | Do not Know | |
| q221oth. Specify other water treatment method  Question relevant when: ${q221} =7 |  |
| q222. What kind of toilet facility does most members of your household use?  - | |  | 1 | Pit Latrine/traditional pit toilet | | --- | --- | --- | |  | 2 | Ventilated improved pit latrine (VIP) | |  | 3 | Flush toilet | |  | 4 | No facility/Bush/Field | |  | 5 | Other | |
| q222oth. Specify other toilet type  Question relevant when: ${q222} =5 |  |
| q223. The last time you passed stool, where did you defecate?  -  Question relevant when: ${q222} !=4 | |  | 1 | Pit Latrine/traditional pit toilet | | --- | --- | --- | |  | 2 | Ventilated improved pit latrine (VIP) | |  | 3 | Flush toilet | |  | 4 | No facility/Bush/Field | |  | 5 | Other | |
| q223oth. Specify other toilet type  Question relevant when: ${q223} =5 |  |
| q225. Main material of the dwelling roof. Record observation.  Main construction material of the roof of the dwelling unit | |  | 1 | Thatch/leaf | | --- | --- | --- | |  | 2 | Rustic mat/plastic sheets | |  | 3 | Reed/bamboo | |  | 4 | Wood planks | |  | 5 | Finished roofing Corrugated iron | |  | 6 | Wood | |  | 7 | Calamine/cement fiber | |  | 8 | Cement/concrete | |  | 9 | Roofing shingles | |  | 10 | Other | |
| q225oth Specify other roof material  Question relevant when: ${q225} =10 |  |
| q226. Main material of the dwelling walls. Record Observation  Main construction material of the walls of the dwelling unit | |  | 1 | No walls | | --- | --- | --- | |  | 2 | Cane/trunks/bamboo/reed | |  | 3 | Rambo/wood | |  | 4 | Stone with mud | |  | 5 | Uncovered adobe | |  | 6 | Plywood | |  | 7 | Cartoon | |  | 8 | Cement | |  | 9 | Stine with lime cement | |  | 10 | Bricks | |  | 11 | Cement blocks | |  | 12 | Covered adobe | |  | 13 | Wood planks/shigles | |  | 14 | Other | |
| q226oth. Specify other wall material  -  Question relevant when: ${q226} =14 |  |
| q227. How many rooms in this household are used for sleeping? (Excluding kitchen and toilets)  Enter 0: if there is one room and they are using it for different purpose; enter 9 if more than 9 rooms  Response constrained to: .<10 |  |
| q228. How many hectar of agricultural land do members of this household own currently?  If none; record “0”. If unknown, record "99". Record in hectar |  |
| q228a. What is the main source of cooking fuel? | |  | 1 | Mainly firewood (purchase or collected) | | --- | --- | --- | |  | 2 | Animal dung | |  | 3 | Crop residue | |  | 4 | Charcoal | |  | 5 | Kerosene | |  | 6 | Butane gas | |  | 7 | Electricity | |  | 8 | Bio gas | |  | 10 | Does not use any cooking fuel or No food cooked in the household | |  | 9 | Other | |
| Specify other  Question relevant when: ${q228a} =9 |  |
| Electricity | |  | 1 | Yes 2. No | | --- | --- | --- | |
| Watches or clocks | |  | 1 | Yes 2. No | | --- | --- | --- | |
| A radio | |  | 1 | Yes 2. No | | --- | --- | --- | |
| A television | | 1 | Yes 2. No | | --- | --- | |
| A mobile telephone | | 1 | Yes 2. No | | --- | --- | |
| A non-mobile telephone | | 1 | Yes 2. No | | --- | --- | |
| A refrigerator | | 1 | Yes 2. No | | --- | --- | |
| A table | | 1 | Yes 2. No | | --- | --- | |
| A chair | | 1 | Yes 2. No | | --- | --- | |
| Mattresses and/or beds | | 1 | Yes 2. No | | --- | --- | |
| An electric mitad | | 1 | Yes 2. No | | --- | --- | |
| A kerosene lamp/pressure lamp | | 1 | Yes 2. No | | --- | --- | |
| Any cattle, sheep, or goats | | 1 | Yes 2. No | | --- | --- | |
| Any jewelry (gold/silver) | | 1 | Yes 2. No | | --- | --- | |
| Information Source  - |  |
| Hours per day  Enter '0' if less than one hour'  Response constrained to: . <25 and . >=0 |  |
| Minutes per day  Question relevant when: ${q230a_hr} =0  Response constrained to: . <60 and . >=0 |  |
| q230b. How much do you rely on television to learn about what is happening in [q101_region] and Ethiopia? Would you say you rely on television not at all, just a bit, or a lot? | |  | 1 | Not at all | | --- | --- | --- | |  | 2 | Just a bit | |  | 3 | A lot | |
| q224. Do you listen to the radio almost every day, at least once a week, less than once a week or not at all? | |  | 1 | Almost every day | | --- | --- | --- | |  | 2 | At least once a week | |  | 3 | Less than once a week | |  | 4 | Not at all | |
| Hours per day  Enter '0' if less than one hour'  Response constrained to: . <25 and . >=0 |  |
| Minutes per day  Question relevant when: ${q230c_hr} =0  Response constrained to: . <60 and . >=0 |  |
| q230d. How much do you rely on radio to learn about what is happening in [q101_region] and Ethiopia? Would you say you rely on radio not at all, just a bit, or a lot? | |  | 1 | Not at all | | --- | --- | --- | |  | 2 | Just a bit | |  | 3 | A lot | |
| Hours per day  Enter '0' if less than one hour'  Response constrained to: . <25 and . >=0 |  |
| Minutes per day  Question relevant when: ${q230e_hr} =0  Response constrained to: . <60 and . >=0 |  |
| q230f. How much do you rely on the mobile phone to learn about what is happening in [q101_region] and Ethiopia? Would you say you rely on the mobile phone not at all, just a bit, or a lot? | |  | 1 | Not at all | | --- | --- | --- | |  | 2 | Just a bit | |  | 3 | A lot | |
| q230h. If you wanted to learn about a new government program designed to help people like you, whom would you turn to for more information?  DO NOT MAKE ANY SUGGESTIONS | |  | a | Spouse or partner | | --- | --- | --- | |  | b | Friend or neighbor | |  | c | Family member or relative (beside spouse or partner) | |  | d | Health extension worker | |  | e | Health Development Army (HDA) person | |  | f | A religious leader | |  | g | Radio | |  | h | TV | |  | i | Telephone /mobile/text | |  | j | Website | |  | k | Newspaper | |  | l | Agricultural development agent | |  | m | Teachers | |  | n | Some other person or media | |  | o | Other sources | |  | p | Do not know | |
| q230goth. Specify other person  Question relevant when: regex( ${q230g} ,'^.*n.*$') |  |
| q230goth2 Specify other source  Question relevant when: regex( ${q230g} ,'^.*o.*$') |  |
| q230h. If you wanted to learn about a new government program to help pregnant mothers, whom would you turn to for more information?  DO NOT MAKE ANY SUGGESTIONS | |  | a | Spouse or partner | | --- | --- | --- | |  | b | Friend or neighbor | |  | c | Family member or relative (beside spouse or partner) | |  | d | Health extension worker | |  | e | Health Development Army (HDA) person | |  | f | A religious leader | |  | g | Radio | |  | h | TV | |  | i | Telephone /mobile/text | |  | j | Website | |  | k | Newspaper | |  | l | Agricultural development agent | |  | m | Teachers | |  | n | Some other person or media | |  | o | Other sources | |  | p | Do not know | |
| q230hoth. Some other person  Question relevant when: regex( ${q230h} ,'^.*n.*$') |  |
| q230hoth2. Other sources  Question relevant when: regex( ${q230h} ,'^.*o.*$') |  |
| q230i. If you wanted to learn about a new and inexpensive soap that they are selling in the market, whom would you turn to for more information?  DO NOT MAKE ANY SUGGESTIONS | |  | a | Spouse or partner | | --- | --- | --- | |  | b | Friend or neighbor | |  | c | Family member or relative (beside spouse or partner) | |  | d | Health extension worker | |  | e | Health Development Army (HDA) person | |  | f | A religious leader | |  | g | Radio | |  | h | TV | |  | i | Telephone /mobile/text | |  | j | Website | |  | k | Newspaper | |  | l | Agricultural development agent | |  | m | Teachers | |  | n | Some other person or media | |  | o | Other sources | |  | p | Do not know | |
| Some other person  Question relevant when: regex( ${q230i} ,'^.*n.*$') |  |
| Other sources  Question relevant when: regex( ${q230i} ,'^.*o.*$') |  |
| If you want to learn about a new agricultural seed for your crops, whom would you turn to for more information?  DO NOT MAKE ANY SUGGESTIONS | |  | a | Spouse or partner | | --- | --- | --- | |  | b | Friend or neighbor | |  | c | Family member or relative (beside spouse or partner) | |  | d | Health extension worker | |  | e | Health Development Army (HDA) person | |  | f | A religious leader | |  | g | Radio | |  | h | TV | |  | i | Telephone /mobile/text | |  | j | Website | |  | k | Newspaper | |  | l | Agricultural development agent | |  | m | Teachers | |  | n | Some other person or media | |  | o | Other sources | |  | p | Do not know | |
| Some other person  Question relevant when: regex( ${q230j} ,'^.*n.*$') |  |
| Other sources  Question relevant when: regex( ${q230j} ,'^.*o.*$') |  |
| If you wanted to learn about a new elementary school opening in your community, whom would you turn to for more information?  DO NOT MAKE ANY SUGGESTIONS | |  | a | Spouse or partner | | --- | --- | --- | |  | b | Friend or neighbor | |  | c | Family member or relative (beside spouse or partner) | |  | d | Health extension worker | |  | e | Health Development Army (HDA) person | |  | f | A religious leader | |  | g | Radio | |  | h | TV | |  | i | Telephone /mobile/text | |  | j | Website | |  | k | Newspaper | |  | l | Agricultural development agent | |  | m | Teachers | |  | n | Some other person or media | |  | o | Other sources | |  | p | Do not know | |
| Some other person  Question relevant when: regex( ${q230k} ,'^.*n.*$') |  |
| Other sources  Question relevant when: regex( ${q230k} ,'^.*o.*$') |  |
| Section 3: Awareness and access to health services |  |
| Have you heard of or do you know about the health extension worker? | | 1 | Yes 2. No | | --- | --- | |
| What are the services provided by the health extension workers?  (Do not prompt; mark items mentioned)  Question relevant when: ${q304} =1 | |  | a | a. Family planning | | --- | --- | --- | |  | b | b. Child immunization | |  | c | c. Antenatal care | |  | d | d. Postnatal care | |  | e | e. Health education | |  | f | f. Growth monitoring | |  | g | g. Referral of sick child | |  | h | h. Diarrhea treatment | |  | i | i. Malaria treatment | |  | j | j. Pneumonia treatment | |  | k | k. Provide or sell bed nets | |  | l | l. Delivery care | |  | m | m. Neonatal care | |  | n | Haygine and sanitation | |  | o | o. Other | |
| q301hph. How many hours does it take to walk to the nearest health post?  enter '0' if less than one hour'; round to the nearest hour; enter 6 if more than 6 hours  Response constrained to: .<7 |  |
| q301hpm. How many minutes does it take to walk to the nearest health post?  Value between 1 and 59 minutes  Question relevant when: ${q301hph} =0  Response constrained to: .<60 and .>0 |  |
| q301hch. How many hours does it take to walk to the nearest health center?  enter '0' if less than one hour'; round to the nearest hour; enter 6 if more than 6 hours  Response constrained to: .<7 |  |
| q301hcm. How many minutes does it take to walk to the nearest health center?  Value between 1 and 59 minutes  Question relevant when: ${q301hch} =0  Response constrained to: .<60 and .>0 |  |
| q302. Have you visited the health post in the last 12 months? | |  | 1 | Yes 2. No | | --- | --- | --- | |  | 8 | Don’t know | |
| q302a. When was the last time you visited the health post?  (between 0 and 11 months) If less than 1 month enter '0'; If don’t know enter 99  Question relevant when: ${q302} =1  Response constrained to: .<12 or .=99 |  |
| q303. The last time you visited the health post, what was the reason for you to visit the health post?  (Do not prompt; mark items mentioned)  Question relevant when: ${q302} =1 | |  | a | a. Family planning | | --- | --- | --- | |  | b | b. Child immunization | |  | c | c. Antenatal care | |  | d | d. Postnatal care | |  | e | e. Health education | |  | f | f. Growth monitoring | |  | g | g. Referral of sick child | |  | h | h. Diarrhea treatment | |  | i | i. Malaria treatment | |  | j | j. Pneumonia treatment | |  | k | k. Provide or sell bed nets | |  | l | l. Delivery care | |  | m | m. Neonatal care | |  | n | Haygine and sanitation | |  | o | o. Other | |
| q303ai. Waiting time for service | |  | 1 | Not satisfied | | --- | --- | --- | |  | 2 | Satisfied | |  | 3 | Very satisfied | |
| q303aii. Ability to discuss your problems, concerns or health issues with providers | |  | 1 | Not satisfied | | --- | --- | --- | |  | 2 | Satisfied | |  | 3 | Very satisfied | |
| q303aiii. Quality of the examination and treatment provided | |  | 1 | Not satisfied | | --- | --- | --- | |  | 2 | Satisfied | |  | 3 | Very satisfied | |
| q303aiv. Privacy from others seeing examination | |  | 1 | Not satisfied | | --- | --- | --- | |  | 2 | Satisfied | |  | 3 | Very satisfied | |
| q303av. Privacy from others hearing discussion | |  | 1 | Not satisfied | | --- | --- | --- | |  | 2 | Satisfied | |  | 3 | Very satisfied | |
| q303avi. Availability of medicine | |  | 1 | Not satisfied | | --- | --- | --- | |  | 2 | Satisfied | |  | 3 | Very satisfied | |
| q303avii. The working hours of service | |  | 1 | Not satisfied | | --- | --- | --- | |  | 2 | Satisfied | |  | 3 | Very satisfied | |
| q303avi1. Cleanliness of the facility | |  | 1 | Not satisfied | | --- | --- | --- | |  | 2 | Satisfied | |  | 3 | Very satisfied | |
| q303b. In general how would you rate the service you received from the health post last time?  Question relevant when: ${q302} =1 | |  | 1 | Not satisfied | | --- | --- | --- | |  | 2 | Satisfied | |  | 3 | Very satisfied | |
| q306. Did the HEW visit your household during the past 6 months to talk about health related issues?  Question relevant when: ${q304} =1 | |  | 1 | Yes 2. No | | --- | --- | --- | |  | 8 | Don’t know | |
| q307. What was discussed or what services were provided by the HEW the last time the HEW visited you at home?  (Do not prompt; mark items mentioned)  Question relevant when: ${q306} =1 | |  | a | a. Message on Immunization | | --- | --- | --- | |  | b | b. Information on child nutrition | |  | c | c. IEC on diarrhea treatment | |  | d | d. Information on pregnancy care | |  | e | e. Information on HIV/AIDS | |  | f | f. Information on hygiene | |  | g | g. Promotion pit latrine construction | |  | h | h. promote latrine use | |  | i | i. promote safe water use | |  | j | j. Information/discussion on Family planning | |  | k | k. Newborn care | |  | l | l. Postnatal care | |  | n | Malaria prevention and control | |  | m | m. Other | |
| q308. Have you heard of or do you know about health development army in your community?  Explain what is an HDA | | 1 | Yes 2. No | | --- | --- | |
| q308a. Are you one of the Health Development Army (HDA) leaders?  -  Question relevant when: ${q308} =1 | |  | 1 | Yes | | --- | --- | --- | |  | 2 | No | |
| If you are one of the HDA leaders, are you a 1:5 or 1:30 leader?  Question relevant when: ${q308a} =1 | |  | 1 | 1:5 leader | | --- | --- | --- | |  | 2 | 1:30 leader | |
| Do you have a map that displays the households under your catchment?  Please observe the map.  Question relevant when: ${q308a1} =2 | |  | 1 | Yes displayed on the wall | | --- | --- | --- | |  | 2 | Yes not displayed on the wall | |  | 3 | Not available | |
| q308b. Are there other HDAs in the neighborhood who have mapped the households in the neighborhood?  Question relevant when: ${q308a1} =1 and ${q308a2} =2 | |  | 1 | Yes 2. No | | --- | --- | --- | |  | 8 | Don’t know | |
| q309. What are the services provided by the health development army (HDA) member?  (Do not prompt; mark items mentioned)  Question relevant when: ${q308} =1 | |  | a | a. Message on Immunization | | --- | --- | --- | |  | b | b. Information on child nutrition | |  | c | c. IEC on diarrhea treatment | |  | d | d. Information on pregnancy care | |  | e | e. Information on HIV/AIDS | |  | f | f. Information on hygiene | |  | g | g. Promotion pit latrine construction | |  | h | h. promote latrine use | |  | i | i. promote safe water use | |  | j | j. Information/discussion on Family planning | |  | k | k. Newborn care | |  | l | l. Postnatal care | |  | n | Malaria prevention and control | |  | m | m. Other | |
| q309oth. Please specify other  Question relevant when: regex( ${q402} ,'^.*m.*$') |  |
| q310. Did any HDA visit you in the home to talk about health related issues during the last six months?  -  Question relevant when: ${q308} =1 and ${q308a} =2 | |  | 1 | Yes | | --- | --- | --- | |  | 2 | No | |
| q310a. When was the last time you were visited by the HDA?  In months, between 0 to 6 months; if less than 1 month enter '0'; if don’t remember enter '98'  Question relevant when: ${q310} =1  Response constrained to: .<7 or .=98 |  |
| q311. What was discussed or what services were provided by the health development army (HDA) the last time she visited you at home?  (Do not prompt; mark items mentioned)  Question relevant when: ${q310} =1 | |  | a | a. Message on Immunization | | --- | --- | --- | |  | b | b. Information on child nutrition | |  | c | c. IEC on diarrhea treatment | |  | d | d. Information on pregnancy care | |  | e | e. Information on HIV/AIDS | |  | f | f. Information on hygiene | |  | g | g. Promotion pit latrine construction | |  | h | h. promote latrine use | |  | i | i. promote safe water use | |  | j | j. Information/discussion on Family planning | |  | k | k. Newborn care | |  | l | l. Postnatal care | |  | n | Malaria prevention and control | |  | m | m. Other | |
| Did you meet any HDA somewhere outside your home to talk about health related issues during the last six months?  -  Question relevant when: ${q308} =1 and ${q308a} =2 | |  | 1 | Yes | | --- | --- | --- | |  | 2 | No | |
| When was the last time you meet the HAD outside your home?  In months, between 0 to 6 months; if less than 1 month enter '0'; if don’t remember enter '98'  Question relevant when: ${q310anyencounter} =1  Response constrained to: .<7 or .=98 |  |
| What was discussed or what services were provided by the HAD during the last encounter outside of your home?  (Do not prompt; mark items mentioned)  Question relevant when: ${q310anyencounter} =1 | |  | a | a. Message on Immunization | | --- | --- | --- | |  | b | b. Information on child nutrition | |  | c | c. IEC on diarrhea treatment | |  | d | d. Information on pregnancy care | |  | e | e. Information on HIV/AIDS | |  | f | f. Information on hygiene | |  | g | g. Promotion pit latrine construction | |  | h | h. promote latrine use | |  | i | i. promote safe water use | |  | j | j. Information/discussion on Family planning | |  | k | k. Newborn care | |  | l | l. Postnatal care | |  | n | Malaria prevention and control | |  | m | m. Other | |
| q311ai. How satified were you by the health issues/topics the HDA discussed with you?  This could be the home visit or any encounter with the HAD  Question relevant when: ${q310} =1 or ${q310anyencounter} =1 | |  | 1 | Not satisfied | | --- | --- | --- | |  | 2 | Satisfied | |  | 3 | Very satisfied | |
| q311aii. How satisfied were you with the manners and respectfulness of the HDA?  Question relevant when: ${q310} =1 or ${q310anyencounter} =1 | |  | 1 | Not satisfied | | --- | --- | --- | |  | 2 | Satisfied | |  | 3 | Very satisfied | |
| q311b01. Does the HDA discuss health issues using books or pictures or is it only verbal communications?  Question relevant when: ${q310} =1 or ${q310anyencounter} =1 | |  | 1 | Only verbal | | --- | --- | --- | |  | 2 | Uses picture or book | |
| q311c01. How often the HDA contacts you to discuss health issues or seek health related information from you?  -  Question relevant when: ${q310} =1 or ${q310anyencounter} =1 | |  | 1 | Every week | | --- | --- | --- | |  | 5 | Every 2 weeks | |  | 2 | Every month | |  | 3 | Every three months | |  | 4 | Less frequent than three months | |  | 99 | Don’t know/don’t remember/non response | |
| q311d01. How satisfied are you with the overall interaction with the HDA?  Question relevant when: ${q310} =1 or ${q310anyencounter} =1 | |  | 1 | Not satisfied | | --- | --- | --- | |  | 2 | Satisfied | |  | 3 | Very satisfied | |
| q312a. Not knowing where to go | |  | 1 | Big problem | | --- | --- | --- | |  | 2 | Small problem | |  | 3 | No problem | |
| q312b. Not Getting permission to go | |  | 1 | Big problem | | --- | --- | --- | |  | 2 | Small problem | |  | 3 | No problem | |
| q312c. Not getting money needed for treatment | |  | 1 | Big problem | | --- | --- | --- | |  | 2 | Small problem | |  | 3 | No problem | |
| q312d. Not having a health facility nearby | |  | 1 | Big problem | | --- | --- | --- | |  | 2 | Small problem | |  | 3 | No problem | |
| q312e. Transportation problem | |  | 1 | Big problem | | --- | --- | --- | |  | 2 | Small problem | |  | 3 | No problem | |
| q312f. Not wanting to go alone | |  | 1 | Big problem | | --- | --- | --- | |  | 2 | Small problem | |  | 3 | No problem | |
| q312g. Concern that there may not be a female health provider | |  | 1 | Big problem | | --- | --- | --- | |  | 2 | Small problem | |  | 3 | No problem | |
| q312h. Concern that there may not be any provider | |  | 1 | Big problem | | --- | --- | --- | |  | 2 | Small problem | |  | 3 | No problem | |
| q312i. due to household chores | |  | 1 | Big problem | | --- | --- | --- | |  | 2 | Small problem | |  | 3 | No problem | |
| q312j. Concern about physical condition of the facility | |  | 1 | Big problem | | --- | --- | --- | |  | 2 | Small problem | |  | 3 | No problem | |
| q312k. Concern about the knowledge of the health care provider | |  | 1 | Big problem | | --- | --- | --- | |  | 2 | Small problem | |  | 3 | No problem | |
| q312l. Concern about the respect shown towards you | |  | 1 | Big problem | | --- | --- | --- | |  | 2 | Small problem | |  | 3 | No problem | |
| q312m. Others will have to take care of children at home | |  | 1 | Big problem | | --- | --- | --- | |  | 2 | Small problem | |  | 3 | No problem | |
| q313a. Not knowing where to go | |  | 1 | Big problem | | --- | --- | --- | |  | 2 | Small problem | |  | 3 | No problem | |
| q313b. Not Getting permission to go | |  | 1 | Big problem | | --- | --- | --- | |  | 2 | Small problem | |  | 3 | No problem | |
| q313c. Not getting money needed for treatment | |  | 1 | Big problem | | --- | --- | --- | |  | 2 | Small problem | |  | 3 | No problem | |
| q313d. Not having a health facility nearby | |  | 1 | Big problem | | --- | --- | --- | |  | 2 | Small problem | |  | 3 | No problem | |
| q313e. Transportation problem | |  | 1 | Big problem | | --- | --- | --- | |  | 2 | Small problem | |  | 3 | No problem | |
| q313f. Not wanting to go alone | |  | 1 | Big problem | | --- | --- | --- | |  | 2 | Small problem | |  | 3 | No problem | |
| q313g. Concern that there may not be a female health provider | |  | 1 | Big problem | | --- | --- | --- | |  | 2 | Small problem | |  | 3 | No problem | |
| q313h. Concern that there may not be any provider | |  | 1 | Big problem | | --- | --- | --- | |  | 2 | Small problem | |  | 3 | No problem | |
| q313i. due to household chores | |  | 1 | Big problem | | --- | --- | --- | |  | 2 | Small problem | |  | 3 | No problem | |
| q313j. Other, specify | |  | 1 | Yes | | --- | --- | --- | |  | 2 | No | |
| q313oth. Specify other problems  Question relevant when: ${q313j} =1 |  |
| Section 4. Community based IEC/BCC: Awareness & expsure |  |
| q401. Have you heard about the Family Health Card? Show Family Health Card  - | |  | 1 | Yes | | --- | --- | --- | |  | 2 | No | |
| q402. If yes, how did you hear about the family health card?  Do not prompt; mark those mentioned  Question relevant when: ${q401} =1 | |  | a | Health Extension Worker | | --- | --- | --- | |  | b | HDAs | |  | c | TBA | |  | f | Health worker | |  | g | Kebele administrators | |  | d | Don’t know | |  | e | Other | |
| Specify other  Question relevant when: regex( ${q402} ,'^.*e.*$') |  |
| q402r. Have you read the FHC or somebody and discussed on it?  -  Question relevant when: ${q401} =1 | |  | 1 | Yes | | --- | --- | --- | |  | 2 | No | |
| q402rw. Who helped you to read and discuss the FHC?  -  Question relevant when: ${q402r} =1 | |  | a | Health Extension Worker | | --- | --- | --- | |  | b | HDAs | |  | c | TBA | |  | f | Health worker | |  | g | Kebele administrators | |  | d | Don’t know | |  | e | Other | |
| q403. Do you/your household have a Family Health Card (FHC)?  -  Question relevant when: ${q401} =1 | |  | 1 | Yes, FHC seen | | --- | --- | --- | |  | 2 | Yes, FHC NOT seen | |  | 3 | No | |
| q403sou. If yes, who gave you the familly health card?  Question relevant when: ${q403} =1 | |  | 1 | HEW | | --- | --- | --- | |  | 2 | HDAs | |  | 3 | Husband | |  | 4 | My children from school | |  | 5 | others | |
| q404. Have you heard about the Immunization Diploma? Show Diploma  - | |  | 1 | Yes | | --- | --- | --- | |  | 2 | No | |
| q405. If yes, how did you hear about the immunization diploma?  Do not prompt; mark those mentioned  Question relevant when: ${q404} =1 | |  | a | Health Extension Worker | | --- | --- | --- | |  | b | HDAs | |  | c | TBA | |  | f | Health worker | |  | g | Kebele administrators | |  | d | Don’t know | |  | e | Other | |
| Specify other  Question relevant when: regex( ${q405} ,'^.*e.*$') |  |
| q406. Do any of your children (under 5 years of age) have immunization diploma?  -  Question relevant when: ${q404} =1 | |  | 1 | Yes, Immunization Diploma seen | | --- | --- | --- | |  | 2 | Yes, Immunization Diploma NOT seen | |  | 3 | No | |  | 4 | Do not have children under 5 years of age | |
| q407. Have you heard about a Model family? | |  | 1 | Yes | | --- | --- | --- | |  | 2 | No | |
| q408. How did you hear about the model family?  (Do not prompt; mark those mentioned)  Question relevant when: ${q407} =1 | |  | a | Health Extension Worker | | --- | --- | --- | |  | b | HDAs | |  | c | TBA | |  | f | Health worker | |  | g | Kebele administrators | |  | d | Don’t know | |  | e | Other | |
| q409. Is this family graduated as a Model Family?  -  Question relevant when: ${q407} =1 | |  | 1 | Yes, graduated (Certificate seen) | | --- | --- | --- | |  | 2 | Yes, graduated (Certificate not seen) | |  | 3 | No, working towards | |  | 4 | Not at all | |
| q409y. If Yes, when did the family graduated as model family?  -  Question relevant when: ${q409} =1 |  |
| q409r. Is the family currently recognized as model family?  Question relevant when: ${q407} =1 | |  | 1 | Yes | | --- | --- | --- | |  | 2 | No | |
| Under 1 children immunized  Question relevant when: ${q407} =1 | |  | 1 | Yes 2. No | | --- | --- | --- | |  | 3 | Not Applicable | |
| Growth monitoring done for children under 2 years  Question relevant when: ${q407} =1 | |  | 1 | Yes | | --- | --- | --- | |  | 2 | No | |  | 3 | Not Applicable | |
| Eligible women used family planning  Question relevant when: ${q407} =1 | |  | 1 | Yes | | --- | --- | --- | |  | 2 | No | |  | 3 | Not Applicable | |
| Eligible women utilize ANC service  Question relevant when: ${q407} =1 | |  | 1 | Yes | | --- | --- | --- | |  | 2 | No | |  | 3 | Not Applicable | |
| Mother delivered at the health facility (Health center or hospital)  Question relevant when: ${q407} =1 | |  | 1 | Yes | | --- | --- | --- | |  | 2 | No | |  | 3 | Not Applicable | |
| Mother received PNC  Question relevant when: ${q407} =1 | |  | 1 | Yes | | --- | --- | --- | |  | 2 | No | |  | 3 | Not Applicable | |
| Mother exclusive breast feed up to 6 months  Question relevant when: ${q407} =1 | |  | 1 | Yes | | --- | --- | --- | |  | 2 | No | |  | 3 | Not Applicable | |
| Frequent use of bed net (where required)  Question relevant when: ${q407} =1 | |  | 1 | Yes | | --- | --- | --- | |  | 2 | No | |  | 3 | Not Applicable | |
| Participate in environmental control for malaria prevention  Question relevant when: ${q409h} !=3 | |  | 1 | Yes | | --- | --- | --- | |  | 2 | No | |  | 3 | Not Applicable | |
| In door residual spraying for malaria and households waited six months to paint their wall again  Question relevant when: ${q409h} !=3 | |  | 1 | Yes | | --- | --- | --- | |  | 2 | No | |  | 3 | Not Applicable | |
| Latrine construction and frequent use  -  Question relevant when: ${q407} =1 | |  | 1 | Yes | | --- | --- | --- | |  | 2 | No | |
| Personal hygiene  -  Question relevant when: ${q407} =1 | |  | 1 | Yes | | --- | --- | --- | |  | 2 | No | |
| Keeping the house clean  -  Question relevant when: ${q407} =1 | |  | 1 | Yes | | --- | --- | --- | |  | 2 | No | |
| Keeping the environment clean (Environmental sanitation)  -  Question relevant when: ${q407} =1 | |  | 1 | Yes | | --- | --- | --- | |  | 2 | No | |
| Separate cooking area  -  Question relevant when: ${q407} =1 | |  | 1 | Yes | | --- | --- | --- | |  | 2 | No | |
| Separate a place for animals and human  -  Question relevant when: ${q407} =1 | |  | 1 | Yes | | --- | --- | --- | |  | 2 | No | |  | 3 | Not Applicable | |
| q410. Do you want your family to be a model family?  Question relevant when: ${q409} =3 or ${q409} =4 | |  | 1 | Yes | | --- | --- | --- | |  | 2 | No | |
| Bed nets |  |
| q501. Does your household have any bed nets that can be used while sleeping? | |  | 1 | Yes | | --- | --- | --- | |  | 2 | No | |  | 3 | Not Applicable | |
| q502. How many mosquito nets does your household have? IF 7 OR MORE NETS, RECORD '7'  if 7 or more nets, record '7'  Question relevant when: ${q501} =1  Response constrained to: .<8 and .>0 |  |
| q503. OBSERVE OR ASK THE BRAND OF THE MOSQUITO NET TO VERIFY BRAND. ASK: When you got the last net, was it already treated with an insecticide to kill or repel mosquitoes?  -  Question relevant when: ${q501} =1 | |  | 1 | Permanent net (Permanent) | | --- | --- | --- | |  | 2 | Pretreated net | |  | 3 | Untreated net | |  | 8 | Do not know | |
| q504. How long ago was the most recent soaking/dipping done? If less than 1 month, record ‘00'.  Enter months; if less than one month enter '0'; if more than 1 year ago enter 95; in don’t know enter '98'  Question relevant when: ${q503} =2  Response constrained to: .<13 or .=95 or .=98 |  |
| q505. Did anyone sleep under mosquito net(s) last night?  Question relevant when: ${q501} =1 | |  | 1 | Yes | | --- | --- | --- | |  | 2 | No | |  | 8 | Don’t know | |
| q506a. Self | |  | 1 | Yes | | --- | --- | --- | |  | 2 | No | |
| q506b. Newborn baby | |  | 1 | Yes | | --- | --- | --- | |  | 2 | No | |
| q506c. Other children (under 5) | |  | 1 | Yes | | --- | --- | --- | |  | 2 | No | |
| q506d. Spouse | |  | 1 | Yes | | --- | --- | --- | |  | 2 | No | |
| q506e. Other | |  | 1 | Yes | | --- | --- | --- | |  | 2 | No | |
| q506oth. Specify other  Question relevant when: ${q506e} =1 |  |
| q507. Did [q104_w0_11] sleep under a bed net last night?  Question relevant when: ${q505} =1 | |  | 1 | Yes | | --- | --- | --- | |  | 2 | No | |
| Section 7: Maternal health |  |
| wq601. Did any community health workers visit you during your pregnancy of [q104_w0_11]? | |  | 1 | Yes | | --- | --- | --- | |  | 2 | No | |  | 8 | Don’t know | |
| q602. Who visited you during your pregnancy? ASK: Who else? Record all responses  -  Question relevant when: ${wq601} =1 | |  | a | Health Extension Worker | | --- | --- | --- | |  | b | HDAs | |  | c | TBA | |  | f | Health worker | |  | g | Kebele administrators | |  | d | Don’t know | |  | e | Other | |
| wq602a. Did the HDA visit you at your home during your pregnancy to talk about health related issues?  Ask this question if the HDA option has not been mentioned in question number 602, if the respondent is not HDA (refer question number 308A but knows the HDA.  Question relevant when: ${wq601} =1 and ${q308a} !=1 | |  | 1 | Yes | | --- | --- | --- | |  | 2 | No | |  | 8 | Don’t know | |
| wq602b. If the HDA didn’t visit you at home; Did you have a chance to encounter with the HDAs in your locality any where in the kebele outside your home to talk about health related issues?  Ask this question if the HDA option has not been mentioned in question number 602, if the respondent is not HDA (refer question number 308A but knows the HDA.)  Question relevant when: ${q308a} =2 | |  | 1 | Yes | | --- | --- | --- | |  | 2 | No | |
| q603. What was discussed during the home or out side home visit/encunter ? ASK: Anything else? Record all responses  Anything else? Record all responses  Question relevant when: ${wq601} =1 or ${wq602b} =1 | |  | a | To get checked up during pregnancy | | --- | --- | --- | |  | b | To get TT vaccination | |  | c | Take Iron Folate tablet | |  | d | To take extra amount of food | |  | e | To take rest | |  | f | To avoid heavy work | |  | g | To seek care if there is a health problem | |  | h | To save money for emergency | |  | i | To arrange for emergency transport | |  | j | To ensure a TBA | |  | k | Put the baby to breast immediately after delivery | |  | l | Give colostrums | |  | m | No pre-lacteals | |  | n | Exclusive breastfeeding | |  | o | LAM | |  | p | Nothing to be applied to the umbilical stump | |  | q | Delay bathing until after 24 hours | |  | r | For you, to sleep under a bed net | |  | s | Counsel and test for HIV | |  | v | Family planning | |  | t | Other (specify) | |  | u | Don’t remember | |
| q603a. While you were pregnant with [q104_w0_11] did any community health worker (HDA or HEW) discuss with you and with your family together at home on pregnancy and child birth?  Question relevant when: ${wq601} =1 | |  | 1 | Yes | | --- | --- | --- | |  | 2 | No | |  | 8 | Don’t know | |
| q603b. Who facilitated the discussion with you and with your family together?  Question relevant when: ${wq603a} =1 | |  | a | Health Extension Worker | | --- | --- | --- | |  | b | HDAs | |  | c | TBA | |  | f | Health worker | |  | g | Kebele administrators | |  | d | Don’t know | |  | e | Other | |
| q603c. Who participated in the discussion at home?  Do not read the response; Ask who else?;<br/>More than 1 response possible  Question relevant when: ${wq603a} =1 | |  | a | Husband | | --- | --- | --- | |  | b | Mother-in-law | |  | c | Mother | |  | d | Sister-in-law | |  | e | Sister | |  | f | Father-in-law | |  | g | Father | |  | h | Neighbor | |  | p | Children (> 15 years of age) | |  | o | Other | |
| Specify other  Question relevant when: regex( ${wq603c} ,'^.*o.*$') |  |
| q603d. What issues were discussed?  Question relevant when: ${wq603a} =1 | |  | a | To get checked up during pregnancy | | --- | --- | --- | |  | b | To get TT vaccination | |  | c | Take Iron Folate tablet | |  | d | To take extra amount of food | |  | e | To take rest | |  | f | To avoid heavy work | |  | g | Where to seek care if there is a health problem | |  | h | Place of delivery | |  | i | How to contact ambulance when necessary | |  | j | How to prepare for facility delivery | |  | k | To save money for emergency | |  | l | To arrange for emergency transport | |  | m | Contact/inform HEW/HDA for assistance with delivery at home | |  | n | Contact/inform HEW/HDA for postnatal care | |  | o | Child Immunization | |  | p | Put the baby to breast immediately after delivery | |  | q | Give colostrums | |  | r | No pre-lacteals | |  | s | Exclusive breastfeeding | |  | t | Nothing to be applied to the umbilical stump | |  | u | Delay bathing until after 24 hours | |  | w | For you, to sleep under a bed net | |  | x | family planning | |  | y | LAM | |  | z | Counsel and test for HIV | |  | za | Don’t remember | |  | oth | Other | |
| q603e. How many times the discussion with you and with your family together occurred at your home?  enter '98' if don’t remember and if > 5 consider as 5  Question relevant when: ${wq603a} =1  Response constrained to: (.<6 and .>0) or .=98 |  |
| q603f. If yes, at what month of pregnancy the first discussion with you and with your family together occurred?  enter '98' if don’t remember; between 2 and 9 months  Question relevant when: ${wq603a} =1  Response constrained to: (.<10 and .>1) or .=98 |  |
| Was there the second session of family conversation?  Question relevant when: ${wq603a} =1 | |  | 1 | Yes | | --- | --- | --- | |  | 2 | No | |
| q603g. If yes, at what month of pregnancy the second discussion with your and with your family together occurred?  enter '98' if don’t remember; between 2 and 9 months  Question relevant when: ${wq603fa} =1  Response constrained to: .> ${wq603f} and .<10 and .>1 or .=98 |  |
| q603g1. Have you ever heard of Pregnant women conference? | |  | 1 | Yes 2.No | | --- | --- | --- | |  | 98 | Don’t know | |
| q603g2. Have you ever participated in Pregnant women conference?  Question relevant when: ${wq603g1} =1 | |  | 1 | Yes 2.No | | --- | --- | --- | |  | 98 | Don’t know | |
| wq603h. During your last pregnancy have you attended a pregnant women's conference/Forum?  Question relevant when: ${wq603g1} =1 | |  | 1 | Yes | | --- | --- | --- | |  | 2 | No | |
| wq603i. If yes, how many times did you attend pregnant women's forum in your last pregnancy?  If greater than 9 times; record as9  Question relevant when: ${wq603h} =1  Response constrained to: .<10 and .>0 |  |
| wq603j. What was discussed in the pregnant women's forum?  Do not read out the list. ASK: Anything else? Record all responses  Question relevant when: ${wq603h} =1 | |  | a | Birth preparedness | | --- | --- | --- | |  | b | Importance of ANC | |  | c | Institutional delivery | |  | d | Importance of post-natal checks | |  | e | Seeking newborn care | |  | g | Immunization | |  | h | Childhood illness | |  | f | Other (specify) | |
| Specify other  Question relevant when: regex( ${wq603j} ,'^.*f.*$') |  |
| wq603k. Who informed you about the pregnant women's forum?  -  Question relevant when: ${wq603h} =1 and ${q308a} =2 | |  | a | HDA | | --- | --- | --- | |  | b | HEW | |  | c | Health worker | |  | d | My husband | |  | f | kebele administratio staff | |  | e | Other | |
| Specify other  Question relevant when: regex( ${wq603k} ,'^.*e.*$') |  |
| wq603l. Where did the last pregnant women conference you attended took place?  Ask about the last one  Question relevant when: ${wq603h} =1 | |  | 1 | At HDA's place | | --- | --- | --- | |  | 2 | At gote/kebele meeting place | |  | 3 | Health post | |  | 4 | Health center | |  | 5 | Kebele administration offcie | |  | 6 | Other | |
| wq603m. Can you tell us whether or not you were satisfied with the pregnant mother conference?  Do not read list of options  Question relevant when: ${wq603h} =1 | |  | 1 | Yes was satisfied | | --- | --- | --- | |  | 2 | No was not satisfied | |  | 3 | Neither satisfied nor dissatisfied | |
| wq603ha. Have you heard/ know that your husband attended any health related meeting/conversation/ forum at kebele level?  Question relevant when: ${q208} =1 | |  | 1 | Yes | | --- | --- | --- | |  | 2 | No | |
| wq603hb. If yes, how many times did he attend such meeting for the last one year?  Question relevant when: ${wq603ha} =1 |  |
| wq603hc. Did your husband attended a meeting while you were pregnant?  Question relevant when: ${wq603ha} =1 | |  | 1 | Yes | | --- | --- | --- | |  | 2 | No | |
| wq603hd. What issues your husband learned from the meeting (he mentioned to you)?  -  Question relevant when: ${wq603ha} =1 | |  | a | Pregnancy related | | --- | --- | --- | |  | b | Delivery related | |  | c | Postpartum related | |  | d | Care to children | |  | e | Feeding to the mother and children | |  | f | Family planning | |  | g | Don't know | |  | h | Other | |
| wq603hdoth. Please specify other  Question relevant when: regex( ${wq603k} ,'^.*h.*$') |  |
| wq603he. What was your condition when your husband attended the meeting?  -  Question relevant when: ${wq603ha} =1 | |  | 1 | Pregnant with ... | | --- | --- | --- | |  | 2 | My child was born | |
| wq603hee. Did your husband discuss with you about the maternal and child health in the last nine months? | |  | 1 | Yes | | --- | --- | --- | |  | 2 | No | |
| wq603hf. What issues did your husband discussed with you in the last nine months?  -  Question relevant when: ${wq603hee} =1 | |  | a | Pregnancy related | | --- | --- | --- | |  | b | Delivery related | |  | c | Postpartum related | |  | d | Care to children | |  | e | Feeding to the mother and children | |  | f | Family planning | |  | g | Don't know | |  | h | Other | |
| wq603hg. If yes, did he use FHG when he discusses health issues with you?  Question relevant when: ${wq603hee} =1 | |  | 1 | Yes | | --- | --- | --- | |  | 2 | No | |
| Now I am going ask you some questions regarding how supportive your husband was in terms of discussing health issues and encouraging you to use MNH services. You are supposed to whether you strongly agree, somewhat agree, not sure, somewhat disagree or strongly disagree.  - |  |
| wq603hi. Your husband encourage you to visit a health facility for pregnancy care  Question relevant when: ${wq603ha} =1 | |  | 1 | Strongly agree | | --- | --- | --- | |  | 2 | Somewhat agree | |  | 3 | Not sure | |  | 4 | Somewhat disagree | |  | 5 | Strongly disagree | |
| wq603hj. Your husband support you to give birth in health facility | |  | 1 | Strongly agree | | --- | --- | --- | |  | 2 | Somewhat agree | |  | 3 | Not sure | |  | 4 | Somewhat disagree | |  | 5 | Strongly disagree | |
| wq603hk.Your husband encourage you to visit health facility for postnatal care | |  | 1 | Strongly agree | | --- | --- | --- | |  | 2 | Somewhat agree | |  | 3 | Not sure | |  | 4 | Somewhat disagree | |  | 5 | Strongly disagree | |
| wq603hl. Your husband support in Seeking propmt medical care in the case of danger signs and complicatons | |  | 1 | Strongly agree | | --- | --- | --- | |  | 2 | Somewhat agree | |  | 3 | Not sure | |  | 4 | Somewhat disagree | |  | 5 | Strongly disagree | |
| wq603hm. Your husband provide you money for health care fees | |  | 1 | Strongly agree | | --- | --- | --- | |  | 2 | Somewhat agree | |  | 3 | Not sure | |  | 4 | Somewhat disagree | |  | 5 | Strongly disagree | |
| wq603hn. Your husband encourage you seek care for your child or himself to take the child to the health facility | |  | 1 | Strongly agree | | --- | --- | --- | |  | 2 | Somewhat agree | |  | 3 | Not sure | |  | 4 | Somewhat disagree | |  | 5 | Strongly disagree | |
| wq603ho. Your husband support you in looking after children or other family member at home whenever you visiy a health faciltiy | |  | 1 | Strongly agree | | --- | --- | --- | |  | 2 | Somewhat agree | |  | 3 | Not sure | |  | 4 | Somewhat disagree | |  | 5 | Strongly disagree | |
| wq603hp. You husband encourage your or supported you to comply with advice or treatment regimen given to you by health professional | |  | 1 | Strongly agree | | --- | --- | --- | |  | 2 | Somewhat agree | |  | 3 | Not sure | |  | 4 | Somewhat disagree | |  | 5 | Strongly disagree | |
| wq603hq. Your husband support you in daily chores at the household level | |  | 1 | Strongly agree | | --- | --- | --- | |  | 2 | Somewhat agree | |  | 3 | Not sure | |  | 4 | Somewhat disagree | |  | 5 | Strongly disagree | |
| wq603hr. Your husband support you to improve your feeding during pregnancy | |  | 1 | Strongly agree | | --- | --- | --- | |  | 2 | Somewhat agree | |  | 3 | Not sure | |  | 4 | Somewhat disagree | |  | 5 | Strongly disagree | |
| wq603hs. Your husband support you to improve your feeding after you gave birth to [q104_w0_11] | |  | 1 | Strongly agree | | --- | --- | --- | |  | 2 | Somewhat agree | |  | 3 | Not sure | |  | 4 | Somewhat disagree | |  | 5 | Strongly disagree | |
| wq603ht. Does your husband encourage you to use family planning after you gave birth to [q104_w0_11] | |  | 1 | Strongly agree | | --- | --- | --- | |  | 2 | Somewhat agree | |  | 3 | Not sure | |  | 4 | Somewhat disagree | |  | 5 | Strongly disagree | |
| wq603hu. Did your husband accompany you to health facility in the last nine month? | |  | 1 | Yes | | --- | --- | --- | |  | 2 | No | |
| wq603hv. What services did you seek care by the time your husband accompanied you to a health facillity?  -  Question relevant when: ${wq603hu} =1 | |  | a | ANC | | --- | --- | --- | |  | b | Delivery | |  | c | PNC | |  | d | EPI | |  | e | FP | |  | f | Care for sick child | |  | g | Others | |
| wq603hw. Have you ever participated in confereences/forums/meetings in which discussions among the groups on health matters were undertaken with the help of audio-video materials in the last nine months (Since January 2009)?  - | |  | 1 | Yes | | --- | --- | --- | |  | 2 | No | |
| wq603hx. If yes, where/when did you watched the audio-video material?  -  Question relevant when: ${wq603hw} =1 | |  | a | During pregnant Women conferences | | --- | --- | --- | |  | b | During community conferences | |  | c | During HDA meetings | |  | d | Maternity waiting homes | |  | e | Others | |
| wq603hy. What topics were discussed using audio-video materials?  -  Question relevant when: ${wq603hw} =1 | |  | a | Danger signs during pregnancy, child birth and PNC | | --- | --- | --- | |  | b | Importance of ANC | |  | c | Birth preparedness and complications readiness | |  | d | Labour and delivery | |  | e | Postnatal care | |  | f | Newborn care | |  | g | Immunization | |  | h | Childhood illness | |
| q604. When you were pregnant with [q104_w0_11] did you go to a health facility for antenatal care? | |  | 1 | Yes | | --- | --- | --- | |  | 2 | No | |  | 8 | Don’t know | |
| q605. How many months pregnant were you when you first received antenatal care for this pregnancy?  enter '98' if don’t remember; between 2 and 9 months  Question relevant when: ${wq604} =1  Response constrained to: (.<10 and .>1) or .=98 |  |
| q606. Where did you receive antenatal care for this pregnancy?  Question relevant when: ${wq604} =1 | |  | a | Hospital | | --- | --- | --- | |  | b | Health Center | |  | c | Health post | |  | d | NGO health facility | |  | e | Private health facility | |  | f | Other | |  | g | Do not know | |
| q607. Who provided antenatal care at the facility? Do not read the responses. Who else?  -  Question relevant when: ${wq604} =1 | |  | a | Doctor | | --- | --- | --- | |  | b | Nurse/midwife | |  | c | Health extension worker | |  | d | Health worker/unknown type | |  | e | Other | |
| q608. How many times did you receive antenatal care in the health facility during this pregnancy?  enter '98' if don’t remember; enter '9' if visited more than 9 times  Question relevant when: ${wq604} =1  Response constrained to: (.<10 and .>0) or .=98 |  |
| wq608a1. How many months pregnant were you when you received your last antenatal care for this pregnancy?  Enter '98' if don't remember; between 2 and 9 months  Question relevant when: ${wq604} =1  Response constrained to: .=98 or (.<10 and .>0) |  |
| wq608a. Please think back to your last (or current) pregnancy. How easy or difficult was (is) it to remember your ANC appointments? Would you say it was very easy, somewhat easy, somewhat difficult, or very difficult?  -  Question relevant when: ${wq608} >1 and ${wq608} <10 | |  | 1 | Very easy | | --- | --- | --- | |  | 2 | Somewhat easy | |  | 3 | Somewhat difficult | |  | 4 | Very difficult | |
| wq608b. How did/do you remember your ANC appointment dates? Who did reminded of your ANC appointment dates?  Question relevant when: ${wq608} >1 and ${wq608} <10 | |  | a | Myself | | --- | --- | --- | |  | b | Using appointment card | |  | c | The HDA | |  | d | The HEW | |  | e | Another health professional | |  | f | A family member | |  | g | A friend | |  | i | My husband | |  | h | Other | |
| Specify other  Question relevant when: regex( ${wq608b} ,'^.*h.*$') |  |
| The next questions are on your ANC experience at the health facility  Question relevant when: ${wq604} =1 |  |
| wq608c. How respectfully were you treated in the health center (or health post)? Would you say very respectfully, respectfully, disrespectfully, or very disrespectfully?  Question relevant when: ${wq604} =1 | |  | 1 | Very respectfully | | --- | --- | --- | |  | 2 | Respectfully | |  | 3 | Disrespectfully | |  | 4 | Very disrespectfully | |
| wq608d. In your opinion, how knowledgeable were the health professionals in the health center (or health post)? Would you say they are very knowledgeable, knowledgeable, not knowledgeable?  Question relevant when: ${wq604} =1 | |  | 1 | Very knowledgeable | | --- | --- | --- | |  | 2 | Knowledgeable | |  | 3 | Not knowledgeable | |
| wq608e.Overall, how comfortable are you at the health center (or health post)? Would you say you are very comfortable, comfortable, uncomfortable, or very uncomfortable?  Question relevant when: ${wq604} =1 | |  | 1 | Very comfortable | | --- | --- | --- | |  | 2 | Comfortable | |  | 3 | Uncomfortable | |  | 4 | Very uncomfortable | |
| wq608f. How responsive was the health center (or health post) to your needs? Would you say it is very responsive, responsive, unresponsive, or very unresponsive?  Question relevant when: ${wq604} =1 | |  | 1 | Very responsive | | --- | --- | --- | |  | 2 | Responsive | |  | 3 | Unresponsive | |  | 4 | Very unresponsive | |
| wq608g. If a close friend of yours were pregnant, would you recommend that she visit that same health center (or health post) where you went for ANC, or would you recommend that she visit another health facility for ANC or would you recommend that she not visit any health facility at all for ANC.  Question relevant when: ${wq604} =1 | |  | 1 | Go to the same health facility | | --- | --- | --- | |  | 2 | Go to another health facility | |  | 3 | Would not recommend that she go to any health facility | |
| Weight Taken | |  | 1 | Yes 2. No | | --- | --- | --- | |  | 8 | Don’t know | |
| Blood pressure Measured | |  | 1 | Yes 2. No | | --- | --- | --- | |  | 8 | Don’t know | |
| Urine Sample Given | |  | 1 | Yes 2. No | | --- | --- | --- | |  | 8 | Don’t know | |
| Blood Sample Given | |  | 1 | Yes 2. No | | --- | --- | --- | |  | 8 | Don’t know | |
| Received Breastfeeding Information | |  | 1 | Yes 2. No | | --- | --- | --- | |  | 8 | Don’t know | |
| Received post partum Family Planning Information | |  | 1 | Yes 2. No | | --- | --- | --- | |  | 8 | Don’t know | |
| Received Information about HIV/AIDS | |  | 1 | Yes 2. No | | --- | --- | --- | |  | 8 | Don’t know | |
| Tested for HIV/ STI | |  | 1 | Yes 2. No | | --- | --- | --- | |  | 8 | Don’t know | |
| Maternal Nutrition Information | |  | 1 | Yes 2. No | | --- | --- | --- | |  | 8 | Don’t know | |
| Given Iron/Folate Supplementation | |  | 1 | Yes 2. No | | --- | --- | --- | |  | 8 | Don’t know | |
| told about danger signs during pregnancy | |  | 1 | Yes 2.No | | --- | --- | --- | |  | 8 | Don’t know | |
| counseled on birth preparedness and complication readiness | |  | 1 | Yes 2. No | | --- | --- | --- | |  | 8 | Don’t know | |
| counseled on neonatal care | |  | 1 | Yes 2. No | | --- | --- | --- | |  | 8 | Don’t know | |
| told about neonatal complications | |  | 1 | Yes 2. No | | --- | --- | --- | |  | 8 | Don’t know | |
| Can you tell us the level of satisfaction or dissatisfaction for the pregnancy care you received at the Health Center/post, Hospital, NGO or Private health facility? | |  | 1 | Fully satisfied | | --- | --- | --- | |  | 2 | Somewhat satisfied | |  | 3 | Somewhat dissatisfied | |  | 4 | Fully dissatisfied | |
| Weight Taken | |  | 1 | Yes 2. No | | --- | --- | --- | |  | 8 | Don’t know | |
| Blood pressure Measured | |  | 1 | Yes 2. No | | --- | --- | --- | |  | 8 | Don’t know | |
| Urine Sample Given | |  | 1 | Yes 2. No | | --- | --- | --- | |  | 8 | Don’t know | |
| Blood Sample Given | |  | 1 | Yes 2. No | | --- | --- | --- | |  | 8 | Don’t know | |
| Given drugs for Malaria | |  | 1 | Yes 2. No | | --- | --- | --- | |  | 8 | Don’t know | |
| Received Breastfeeding Information | |  | 1 | Yes 2. no | | --- | --- | --- | |  | 8 | Don’t know | |
| Received Family Planning Information | |  | 1 | Yes 2. No | | --- | --- | --- | |  | 8 | Don’t know | |
| Received Information about HIV/AIDS | |  | 1 | Yes 2. No | | --- | --- | --- | |  | 8 | Don’t know | |
| Tested for HIV/ STI | |  | 1 | Yes 2. No | | --- | --- | --- | |  | 8 | Don’t know | |
| Maternal Nutrition Information | |  | 1 | Yes 2. No | | --- | --- | --- | |  | 8 | Don’t know | |
| Given Iron/Folate Supplementation | |  | 1 | Yes 2. No | | --- | --- | --- | |  | 8 | Don’t know | |
| told about danger signs during pregnancy | |  | 1 | Yes 2. No | | --- | --- | --- | |  | 8 | Don’t know | |
| counseled on birth preparedness and complication readiness | |  | 1 | Yes 2. No | | --- | --- | --- | |  | 8 | Don’t know | |
| counseled on neonatal care | |  | 1 | Yes | | --- | --- | --- | |  | 8 | Don’t know | |
| told about neonatal complications | |  | 1 | Yes 2. No | | --- | --- | --- | |  | 8 | Don’t know | |
| Can you tell us the level of satisfaction or dissatisfaction for the pregnancy care you received at the Health post? | |  | 1 | Fully satisfied | | --- | --- | --- | |  | 2 | Somewhat satisfied | |  | 3 | Somewhat dissatisfied | |  | 4 | Fully dissatisfied | |
| q610. During this pregnancy, were you given an injection in the arm to prevent the baby from getting tetanus, that is, convulsions after birth?  - | |  | 1 | Yes 2. No | | --- | --- | --- | |  | 8 | Don’t know | |
| q611. During this pregnancy, how many times did you get this tetanus injection?  enter '98' if don’t remember  Question relevant when: ${wq610} =1  Response constrained to: (.<4 and .>0) or .=98 |  |
| q612. At any time before this pregnancy with [q104_w0_11] , did you receive any tetanus injections? | |  | 1 | Yes 2. No | | --- | --- | --- | |  | 8 | Don’t know | |
| q613. Before this pregnancy, how many other times did you receive a tetanus injection?  enter '98' if don’t remember  Question relevant when: ${wq612} =1  Response constrained to: (.<10 and .>0) or .=98 |  |
| q614. How many years ago did you receive the last tetanus injection before this pregnancy?  enter '98' if don’t remember  Question relevant when: ${wq613} >0 and ${wq613} <98  Response constrained to: (.<20 and .>0) or .=98 |  |
| q615. When you were pregnant with [q104_w0_11] did you eat, less than usual, the same as usual, or more than usual?  - | |  | 1 | Less | | --- | --- | --- | |  | 2 | Same | |  | 3 | More | |  | 8 | Don’t Know | |
| q616. When you were pregnant with [q104_w0_11] did you regularly take iron tablets? | |  | 1 | Yes | | --- | --- | --- | |  | 2 | No | |  | 8 | Don’t know | |
| q617. For how many months did you take iron tablets regularly when you were pregnant with [q104_w0_11]?  enter '98' if don’t remember; '0' if less than 1 month  Question relevant when: ${wq616} =1  Response constrained to: .<9 or .=98 |  |
| q619. When you were pregnant with [q104_w0_11] did you take any drugs for intestinal parasite? | |  | 1 | Yes | | --- | --- | --- | |  | 2 | No | |  | 8 | Don’t know | |
| q620. While you were pregnant with [q104_w0_11], did any health worker/HEW discuss with you about using family planning method after giving birth? | |  | 1 | Yes | | --- | --- | --- | |  | 2 | No | |  | 8 | Don’t know | |
| Birth Preparedness |  |
| q701. During pregnancy of [q104_w0_11] did you make any preparations for your delivery?  Only if necessary tell to the respondent that may include financial, who would attend, where to deliver, transportation, etc | |  | 1 | Yes | | --- | --- | --- | |  | 2 | No | |
| q702. What preparations did you make for the delivery of [q104_w0_11]?  Do not read responses, ASK: Anything else? Record all responses  Question relevant when: ${wq701} =1 | |  | a | Financial | | --- | --- | --- | |  | b | Transport | |  | c | Food | |  | d | Arrange birth attendants | |  | e | Identified health facility for delivery | |  | f | Prepared clean and appropriate materials for delivery | |  | g | Identified blood donors | |  | i | Arrange a person who will care for the children | |
| q702a. What kind of transport mechanism you prepared for your deliver?  Question relevant when: ${wq701} =1 and selected( ${wq702} ,'b') | |  | a | Gari | | --- | --- | --- | |  | b | Animals (horse, mule) | |  | c | Stretcher | |  | d | Ambulance | |  | e | Public transport | |  | f | Government office vehicle | |  | g | Walk | |  | i | Carried by relatives | |  | h | Other | |
| q703. Who did you plan to attend [q104_w0_11]'s delivery? Do not read responses.  ASK: Anything else? Record all responses  Question relevant when: ${wq701} =1 | |  | a | Untrained TBA | | --- | --- | --- | |  | c | Mother | |  | d | Mother-in-law | |  | e | Other female relative | |  | f | Health Extension Worker | |  | g | Community health volunteers(WDTs) | |  | h | Skilled (doctor/nurse/midwife) | |  | o | Other | |
| q704. Did you plan for a place to deliver [q104_w0_11]?  Question relevant when: ${wq701} =1 | |  | 1 | Yes | | --- | --- | --- | |  | 2 | No | |
| q704a. While you were pregnant with [q104_w0_11] did any health worker/HDA discuss with you and with your family together to decide a place of deliver?  Question relevant when: ${wq704} =1 | |  | 1 | Yes | | --- | --- | --- | |  | 2 | No | |
| q705. Where did you plan to deliver [q104_w0_11]?  -  Question relevant when: ${wq704} =1 | |  | 1 | My Home | | --- | --- | --- | |  | 2 | Other Home | |  | 3 | Government Hospital | |  | 4 | Government Health Center | |  | 5 | Health post | |  | 6 | NGO Health Facility | |  | 7 | Private Hospital | |  | 8 | Private Doctor/Clinic | |  | 9 | Other | |
| q706. Who was the main person decided where [q104_w0_11]’s delivery should take place?  Question relevant when: ${wq704} =1 | |  | 1 | Self | | --- | --- | --- | |  | 2 | Mother-in-law | |  | 3 | Father-in-law | |  | 4 | Other relative | |  | 5 | My husband | |  | 6 | My mother | |  | 11 | HEW | |  | 12 | HDA | |  | 13 | I and my husband | |  | 10 | Other | |
| Sometimes mothers during pregnacy have severe illnesses and should be taken immediately to a health facility. What type of symptoms would cause mothers to go to a health facility right away?  Do not read responses; ASK: Anything else?; Record all responses | |  | a | Severe headache | | --- | --- | --- | |  | b | Blurred vision | |  | c | Reduced fetal movement | |  | d | Unconsciousness | |  | e | Convulsions | |  | f | Vaginal bleeding | |  | i | Severe lower abdominal pain | |  | j | Fever | |  | g | Swollen hands and face | |  | h | No symptom mentioned | |
| Did you have any of the symptoms of the danger signs of pregnancy when pregnant with [q104_w0_11]? | |  | 1 | Yes | | --- | --- | --- | |  | 2 | No | |
| Which of the symptoms of the danger signs of pregnancy did you have when pregnant with [q104_w0_11]?  -  Question relevant when: ${wq941} =1 | |  | a | Severe headache | | --- | --- | --- | |  | b | Blurred vision | |  | c | Reduced fetal movement | |  | d | Unconsciousness | |  | e | Convulsions | |  | f | Vaginal bleeding | |  | i | Severe lower abdominal pain | |  | j | Fever | |  | g | Swollen hands and face | |  | h | No symptom mentioned | |
| What did you do when you had any of the danger signs?  Question relevant when: ${wq941} =1 | |  | a | Hospital | | --- | --- | --- | |  | b | Health center | |  | c | Health post | |  | d | Drug shop/pharmacy | |  | f | Health development army leaders (HDAs) | |  | g | TBA/TTBA | |  | h | Holy water/religious place | |  | i | Traditional healer/Witchcraft | |  | j | Other(specify) | |
| Delivery and newborn care |  |
| wq801. Where did you give birth to [q104_w0_11]? | |  | 1 | My Home | | --- | --- | --- | |  | 2 | Other Home | |  | 3 | Government Hospital | |  | 4 | Government Health Center | |  | 5 | Health post | |  | 6 | NGO Health Facility | |  | 7 | Private Hospital | |  | 8 | Private Doctor/Clinic | |  | 9 | Other | |
| If delivered at home, why?  Select all that apply  Question relevant when: ${wq801} =1 or ${wq801} =2 | |  | a | Always delivered at home | | --- | --- | --- | |  | b | Husband/mother/mother-in-law does not allow | |  | c | Don't like health facilities | |  | d | Expensive to go to health facilities | |  | g | Other cost (not medical cost) | |  | h | Facility not open | |  | e | Tradition/religious reason | |  | i | Poor quality service | |  | j | No female provider | |  | k | Not necessary | |  | l | Not customary | |  | f | Other | |
| If delivered in health facility why?  Select all that apply  Question relevant when: ${wq801} =3 or ${wq801} =4 or ${wq801} =5 or ${wq801} =6 or ${wq801} =7 or ${wq801} =8 | |  | a | Always delivered at a health facility | | --- | --- | --- | |  | b | Due to existing complications | |  | c | Referred by HDA/HEW | |  | d | Advised at pregnant women's forum | |  | e | Taken to health facility due to prolonged labour or delivery related risks | |  | f | Convenient | |  | g | Affordable (free) | |  | i | Advised by HDAs | |  | j | Advised during ANC | |  | h | Other | |
| wq801a. For your last pregnancy, were you given an estimated delivery date during your antenatal check-up?  Question relevant when: ${wq604} =1 | |  | 1 | Yes | | --- | --- | --- | |  | 2 | No | |
| wq801b. Who provided the estimated delivery dates?  Question relevant when: ${wq801a} =1 | |  | 1 | Doctor | | --- | --- | --- | |  | 2 | Nurse/Midwife | |  | 3 | Health extension worker | |  | 4 | Health worker (unknown type) | |  | 5 | Other (specify) | |
| wq801c. Did you deliver on the estimated delivery date, before that date, or earlier than that date?  Question relevant when: ${wq801a} =1 | |  | 1 | On the delivery date | | --- | --- | --- | |  | 2 | Before delivery date | |  | 3 | After delivery date | |
| wq800a. Did you or anybody in your family or neighborhood notify the health worker/health facility/ambulance regarding onset of your labor for birthing [q104_w0_11]? | |  | 1 | Yes | | --- | --- | --- | |  | 2 | No | |  | 8 | Don’t know | |  | 9 | Stays at MWH | |
| q800b. For whom did you or anybody in your family or neighborhood notify the onset of your labor ?  Question relevant when: ${wq800a} =1 | |  | a | HEW | | --- | --- | --- | |  | b | HDA | |  | c | Ambulance | |  | d | Other | |
| Specify other  Question relevant when: regex( ${wq800b} ,'^.*d.*$') |  |
| wq800d. If Yes, who notified the birth?  Question relevant when: ${wq800a} =1 | |  | 1 | Your husband | | --- | --- | --- | |  | 2 | HDAs | |  | 3 | Your children | |  | 4 | Neghbour | |  | 5 | Other | |
| wq800e. What mechanism ddi you use to notify your birth? | |  | 1 | Phone call | | --- | --- | --- | |  | 2 | Send a card | |  | 3 | Send messenger | |  | 4 | Other | |
| Specify other  Question relevant when: ${wq800e} =4 |  |
| q801d. What type of transportation means you used to reach to the place you delivered?  Do not read responses, ASK: Anything else? Record all responses. Note:- this question will be asked if the mother only delivered at health facility (hospital, health Center, health Station/Clinic, NGO Health Facility, private Doctor/Clinic)  Question relevant when: ${wq801} =3 or ${wq801} =4 or ${wq801} =5 or ${wq801} =7 or ${wq801} =8 or ${wq801} =9 | |  | a | Gari | | --- | --- | --- | |  | b | Animals (horse, mule) | |  | c | Stretcher | |  | d | Ambulance | |  | e | Public transport | |  | f | Government office vehicle | |  | g | Walk | |  | i | Carried by relatives | |  | h | Other | |
| How did you inform to get the ambulance?  Ask the main person  Question relevant when: regex( ${wq801d} ,'^.*d.*$') | |  | 1 | Myself | | --- | --- | --- | |  | 2 | My husband | |  | 3 | Other family member | |  | 4 | The WDT | |  | 5 | The HEW | |  | 6 | The Kebele administrators | |  | 7 | The women association head | |  | 8 | Other | |
| Specify other  Question relevant when: ${wq801d1} =8 |  |
| For how long you waited to get the ambulance after the inforamtion? (Hours)  If less than 1 hour record '0', if greater than 24 hours put 24 and put 98 if do not remember  Question relevant when: regex( ${wq801d} ,'^.*d.*$')  Response constrained to: .<=24 or .=98 |  |
| For how many minutes you waited to get the ambulance after the inforamtion?  Question relevant when: ${wq801d2} =0  Response constrained to: .<60 and .>0 or .=98 |  |
| How much reasonable is the wait?  Question relevant when: regex( ${wq801d} ,'^.*d.*$') | |  | 1 | Reasonable | | --- | --- | --- | |  | 2 | Too long 3. Don’t know | |
| What type of transportation means you used to go back to home?  Do not read responses, ASK: Anything else? Record all responses. Note:- this question will be asked if the mother only delivered at health facility (hospital, health Center, health Station/Clinic, NGO Health Facility, private Doctor/Clinic)  Question relevant when: ${wq801} =3 or ${wq801} =4 or ${wq801} =5 or ${wq801} =7 or ${wq801} =8 or ${wq801} =9 | |  | a | Gari | | --- | --- | --- | |  | b | Animals (horse, mule) | |  | c | Stretcher | |  | d | Ambulance | |  | e | Public transport | |  | f | Government office vehicle | |  | g | Walk | |  | i | Carried by relatives | |  | h | Other | |
| q802. Who was the primary person that assisted you with the delivery of [q104_w0_11]?  - | |  | 1 | Doctor | | --- | --- | --- | |  | 2 | Nurse/midwife | |  | 3 | Health extension worker | |  | 9 | Health worker (unknown type) | |  | 4 | HAD | |  | 5 | traditional birth attendant | |  | 6 | Relative/friend | |  | 7 | Nobody | |  | 8 | Other(specify) | |
| When you gave birth, did the main person assisting you wash her/his hands with soap before the delivery?  - | |  | 1 | Yes 2. No | | --- | --- | --- | |  | 8 | Don’t know | |
| When you gave birth, did the main person assisting you wear gloves during delivery? | |  | 1 | Yes 2. No | | --- | --- | --- | |  | 8 | Don’t know | |
| When you gave birth, did the delivery take place on a clean surface? (Clean surface: clean space or carpet or mat)  (Clean surface: clean space or carpet or mat) | |  | 1 | Yes | | --- | --- | --- | |  | 2 | No | |  | 8 | Don’t know | |
| q803. Where you given any drugs(misoprostol)/injection to prevent excessive bleeding after giving birth to [q104_w0_11]?  - | |  | 1 | Yes 2. No | | --- | --- | --- | |  | 8 | Don’t know | |
| q802a. Who else was present at the delivery?  Do not read responses; ASK Anything else? Probe for the type of person and record all persons assisting | |  | a | Doctor | | --- | --- | --- | |  | b | Nurse/midwife | |  | c | Health extension worker | |  | d | HAD | |  | e | traditional birth attendant | |  | f | Relative/friend | |  | g | Nobody | |  | h | Other(specify) | |
| Was [q104_w0_11] delivered by caesarean, that is, did they cut your belly open to take the baby out?  Question relevant when: ${wq801} =3 or ${wq801} =4 or ${wq801} =6 or ${wq801} =7 or ${wq801} =8 | |  | 1 | Yes | | --- | --- | --- | |  | 2 | No | |
| wq803a. After the delivery of [q104_w0_11], did any health worker at the health facility you delivered discussed with you about family planning method that can be adopted after giving birth?  Question relevant when: ${wq801} =5 or ${wq801} =3 or ${wq801} =4 or ${wq801} =7 or ${wq801} =8 or ${wq801} =9 | |  | 1 | Yes | | --- | --- | --- | |  | 2 | No | |
| wq803b. Have you or your husband ever used any method to delay or avoid getting pregnant after [q104_w0_11] was born? | |  | 1 | Yes | | --- | --- | --- | |  | 2 | No | |
| wq803d. Are you or your partner currently doing something or using any method to delay or avoid getting pregnant?  Question relevant when: ${wq803b} =1 | |  | 1 | Yes | | --- | --- | --- | |  | 2 | No | |
| wq803e. Which method are you currently using?  Do not read out responses  Question relevant when: ${wq803d} =1 | |  | 1 | Female Sterilization | | --- | --- | --- | |  | 2 | Male Sterilization | |  | 3 | Pill | |  | 4 | IUD | |  | 5 | Injections | |  | 6 | Implants | |  | 7 | Condom | |  | 8 | Diaphragm/Foam/Jelly | |  | 9 | Periodic Abstinence | |  | 10 | Withdrawal | |  | 11 | LAM | |  | 13 | Do not know | |  | 14 | Emergency contraception | |  | 15 | Standard days method/Cycle Beads | |  | 12 | Other | |
| wq803f. Where did you obtain (CURRENT METHOD) the last time?  Do not read out responses  Question relevant when: ${wq803d} =1 | |  | 1 | Government hospital | | --- | --- | --- | |  | 2 | Government health center | |  | 3 | Other Government health facility | |  | 4 | Government health post | |  | 5 | Government Outreach | |  | 6 | NGO Health facility | |  | 7 | NGO Outreach | |  | 8 | Private Hospital | |  | 9 | Private doctor/clinic | |  | 10 | Pharmacy | |  | 11 | Drug Vendor | |  | 12 | Kiosk | |  | 13 | Friend/Relative | |  | 14 | Don’t know/don’t remember | |  | 15 | Other | |
| The next questions are on your delivery experience at the health facility  Question relevant when: ${wq801} =3 or ${wq801} =4 or ${wq801} =5 or ${wq801} =7 or ${wq801} =8 or ${wq801} =10 |  |
| In your opinion, how knowledgeable are the health professionals in the health center? Would you say they are very knowledgeable, knowledgeable, and not knowledgeable?  Question relevant when: ${wq801} =3 or ${wq801} =4 or ${wq801} =5 or ${wq801} =7 or ${wq801} =8 or ${wq801} =10 | |  | 1 | Very knowledgeable | | --- | --- | --- | |  | 2 | Knowledgeable | |  | 3 | Not knowledgeable | |
| wq803k. If a close friend of yours were pregnant, would you recommend that she deliver at the same health center where you did, would you recommend that she delivery at another health center or would you recommend that she not deliver at any health facility?  Question relevant when: ${wq801} =3 or ${wq801} =4 or ${wq801} =5 or ${wq801} =7 or ${wq801} =8 or ${wq801} =10 | |  | 1 | Go to the same health facility | | --- | --- | --- | |  | 2 | Go to another health facility | |  | 3 | Would not recommend that she go to any health facility | |
| Maternity waiting home  Question relevant when: ${wq801} =3 or ${wq801} =4 |  |
| Did you go to the Health center or Hospital before having labor/delivery pain and stay there?  Question relevant when: ${wq801} =3 or ${wq801} =4 | |  | 1 | Yes | | --- | --- | --- | |  | 2 | No | |
| Where did you stay?  Ask the main place where they stayed  Question relevant when: ${wq801} =3 or ${wq801} =4 and ${wq801mwh1} =1 | |  | 1 | Relatives house | | --- | --- | --- | |  | 2 | Hotel | |  | 3 | Maternity waiting homes | |  | 4 | Other | |
| Who advised you to go to the maternity waiting home?  Question relevant when: ${wq801mwh2} =3 | |  | a | HEW | | --- | --- | --- | |  | b | WDT/HAD | |  | c | Health workers from hospital | |  | d | Health workers from health center | |  | e | Advised at pregnant women's forum | |  | f | Other | |
| Are you aware that women can go the MWH before labor and wait for childbirth?  -  Question relevant when: ${wq801mwh2} !=3 | |  | 1 | Yes | | --- | --- | --- | |  | 2 | No | |
| If not utilized the maternity waiting home, why did you not go there?  Question relevant when: ${wq801mwh2} !=3 and ${wq801mwh4} =1 | |  | a | Facility is very near | | --- | --- | --- | |  | b | No one told me to stay at the waiting homes | |  | c | Not knowing due date (EDD) | |  | d | Husban/family does not allow | |  | e | Not important | |  | f | No one to care children/family at home | |  | g | No one to care/accompany me | |  | i | Lack of transport | |  | j | The MWH is not clean | |  | k | Tradition/religious reason | |  | l | Other | |
| Did you make the decision yourself or jointly with family and/or provider to come to the waiting home?  Question relevant when: ${wq801mwh2} =3 | |  | 1 | Myself | | --- | --- | --- | |  | 2 | Jointly with family | |  | 3 | Jointly with provider or community health worker | |  | 4 | other | |
| If advised, by health facility or community worker? Who advised you to go there?  Question relevant when: ${wq801mwh6} =3 | |  | 1 | Facility | | --- | --- | --- | |  | 2 | Community | |
| For how many days you stayed there before your delivery?  Question relevant when: ${wq801mwh2} =3  Response constrained to: .<60 |  |
| Did the health worker conduct any check-up after you arrived at the maternity waiting home?  Question relevant when: ${wq801mwh2} =3 | |  | 1 | Yes | | --- | --- | --- | |  | 2 | No | |
| Did you stay at the MWH after delivery?  Question relevant when: ${wq801mwh2} =3 | |  | 1 | Yes | | --- | --- | --- | |  | 2 | No | |
| How long after delivery you stayed there? (Hours)  If stayed more than a week record as 168 hours (7days)  Question relevant when: ${wq801mwh9a} =1  Response constrained to: .<169 |  |
| If your neighbor were pregnant, would you recommend that she comes to this waiting home?  Question relevant when: ${wq801mwh2} =3 | |  | 1 | Yes | | --- | --- | --- | |  | 2 | No | |
| Can you tell us whether or not you were satisfied with the service you received from the maternity waiting homes?  Question relevant when: ${wq801mwh2} =3 | |  | 1 | Fully satisfied | | --- | --- | --- | |  | 2 | Somewhat satisfied | |  | 3 | Somewhat dissatisfied | |  | 4 | Fully dissatisfied | |
| If not satisfied, or if would not recommend to other women, then why not?  Question relevant when: ${wq801mwh2} =3 and ${wq801mwh12} =3 or ${wq801mwh12} =4 or ${wq801mwh11} =2 | |  | a | Health workers treatment was not good (No respect) | | --- | --- | --- | |  | b | It has no home like environment | |  | c | Not convenient to prepare food | |  | d | Don't like the physical structure of the house | |  | e | Too many mothers(crowded) | |  | f | Size of the room is not adequate | |  | g | Don't like the bed | |  | h | Not comfortable to share room with others | |  | i | Have other children those needs care at home | |  | j | Lack of privacy | |  | k | lack of health care | |  | l | Not clean | |  | n | Lack of food supply | |  | o | There are no recreational opportunities (No TV or radio or video) | |  | m | Other | |
| Specify other  Question relevant when: regex( ${wq801mwh13} ,'^.*m.*$') |  |
| wq804imm. Did you initiate breastfeeding immediately after birth?  Initiated within one hour? | |  | 1 | Yes | | --- | --- | --- | |  | 2 | No | |
| Hours after birth  enter '0 ' if less than 1 hour; enter '66' if after the 1st day (if >24); enter '98' if don’t know or did not initiate breastfeeding  Question relevant when: ${wq804imm} =1  Response constrained to: .<25 or .=66 or .=98 |  |
| Days after birth  If after more than 24 hours; between 1 and 30 days; enter '98' if more than 30 days; enter '99' if don’t remember  Question relevant when: ${wq804h} =66  Response constrained to: (.<31 and .>0) or .=99 or .=98 |  |
| q805. What did you do with the first milk? | |  | 1 | Squeeze out and throw | | --- | --- | --- | |  | 2 | Squeeze out and give to the baby | |  | 8 | Other/don’t remember | |
| q806. Was [q104_w0_11] weighed at birth? | |  | 1 | Yes 2. No | | --- | --- | --- | |  | 8 | Don’t know | |
| q807a. How much did [q104_w0_11] weigh?  from card; in Kg; between 1 and 6 kg; enter 99.99 if no card  Question relevant when: ${wq806} =1  Response constrained to: (.<7 and .>0 ) or .=99.99 |  |
| q807minf. How much did [q104_w0_11] weigh?  from recall; in Kg; between 1 and 6 kg; enter 99.99 if don’t know  Question relevant when: ${wq806} =1 and ${wq807a} =99.99  Response constrained to: (.<7 and .>0 ) or .=99.99 |  |
| q808. When [q104_w0_11] was born, was he/she very large, larger than average, average, smaller than average, or very small? | |  | 1 | Large | | --- | --- | --- | |  | 2 | Average | |  | 3 | Small | |  | 4 | Born too early | |  | 8 | Don’t know | |
| q809. When [q104_w0_11] was delivered, what instrument was used to cut the cord?  Question relevant when: ${wq801} =1 or ${wq801} =2 | |  | 1 | New Blade | | --- | --- | --- | |  | 2 | Boiled Blade | |  | 3 | Unboiled used blade | |  | 4 | Knife | |  | 5 | Scissor | |  | 6 | Do not know | |  | 7 | Other (specify) | |
| q810. When [q104_w0_11] was delivered, what was used to tie the cord?  Question relevant when: ${wq801} =1 or ${wq801} =2 | |  | 1 | New string or thread | | --- | --- | --- | |  | 2 | Boiled string or thread | |  | 3 | Used string or thread | |  | 4 | Did not tie the cord | |  | 8 | Do not know | |  | 111 | Other (specify) | |
| q811. When [q104_w0_11] was delivered, did anybody apply anything on the stump after the baby’s cord was cut? | |  | 1 | Yes 2. No | | --- | --- | --- | |  | 8 | Don’t know | |
| q812. If so, what did they apply?  Do not read responses; ASK: Anything else?; Record all responses  Question relevant when: ${wq811} =1 | |  | a | Butter | | --- | --- | --- | |  | b | Oil | |  | c | Ash | |  | d | Ointment/powder | |  | e | Animal dung | |  | f | Cold water | |  | h | Chlorhexidine | |  | g | Other/don’t remember | |
| Was chlorhexidine applied to the cord?  Question relevant when: ${wq811} =1 | |  | 1 | Yes 2. No | | --- | --- | --- | |  | 8 | Don’t know | |
| If YES, for how many days was chlorhexidine applied?  -  Question relevant when: ${wq811} =1 and ${wq812a} =1  Response constrained to: .<10 or .=99 |  |
| Did [q104_w0_11] receive TTC eye ointment soon after delivery? | |  | 1 | Yes 2. No | | --- | --- | --- | |  | 8 | Don’t know | |
| q813. Was your baby [q104_w0_11] dried/wiped before the placenta was delivered or immediately after birth? | |  | 1 | Yes 2. NO | | --- | --- | --- | |  | 8 | Don’t know | |
| How long after [q104_w0_11] was born was s/he dried/wiped?  Record in hours; enter '0' if immediately, if more than 3 days enter 72; if don’t remember enter '99'  Question relevant when: ${wq813} =1  Response constrained to: .<73 or .=99 |  |
| q814. Was your baby [q104_w0_11] wrapped in cloth before the placenta was delivered or immediately after birth? | |  | 1 | Yes 2. No | | --- | --- | --- | |  | 8 | Don’t know | |
| How long after [q104_w0_11] was born was s/he wrapped in a cloth?  Record in hours; enter '0' if immediately, if more than 3 days enter 72; if don’t remember enter '99'. Check for time after the baby was born, not time after the placenta came out.  Question relevant when: ${wq814} =1  Response constrained to: .<73 or .=99 |  |
| q815. Was your baby [q104_w0_11] put on the breast before the placenta was delivered or immediately after birth? | |  | 1 | Yes 2. No | | --- | --- | --- | |  | 8 | Don’t know | |
| q816. Where was the baby [q104_w0_11] placed before the placenta was delivered or immediately after birth? In Hours | |  | 1 | Alone/on the floor | | --- | --- | --- | |  | 2 | On the mother’s Belly/chest | |  | 3 | Beside the mother | |  | 4 | With someone else | |  | 5 | On the cot | |  | 7 | Don’t know | |  | 6 | Other | |
| q817. Did the baby [q104_w0_11] cry or breath easily immediately after birth? | |  | 1 | Yes 2. No | | --- | --- | --- | |  | 8 | Don’t know | |
| q818. What was done to help the baby [q104_w0_11] cry or breath easily immediately after birth?  Do not read responses; ASK: Anything else?; Record all responses  Question relevant when: ${wq817} =2 | |  | a | Rubbed/massaged/Stimulating | | --- | --- | --- | |  | b | Dried | |  | c | Mouth cleared | |  | d | Mouth to mouth resuscitation | |  | e | Ambu bag used | |  | f | Nothing | |  | i | Suction | |  | h | Don't know | |  | g | Other | |
| q819. Who took these measures to help the baby [q104_w0_11] cry or breathed?  Question relevant when: ${wq817} =2 | |  | 1 | Health worker | | --- | --- | --- | |  | 2 | Health Extension Worker | |  | 3 | HDA | |  | 4 | Traditional birth attendant | |  | 7 | Family/relative/friend | |  | 8 | Self | |  | 9 | Other | |
| q820. How long after birth was your baby [q104_w0_11] bathed for the first time?  Record in hours; if more than 3 days enter 72; if don’t remember enter '98'  Response constrained to: .<73 or .=98 |  |
| q821. In the first three days after delivery, was the baby [q104_w0_11] given anything to drink other than breast milk? | |  | 1 | Yes 2. No | | --- | --- | --- | |  | 8 | Don’t know | |
| q822. What was the baby [q104_w0_11] given to drink?  Do not read responses; ASK: Anything else?; Record all responses  Question relevant when: ${wq821} =1 | |  | a | Milk (other than breast milk) | | --- | --- | --- | |  | b | Plain water | |  | c | Sugar or glucose water | |  | d | Fruit juice | |  | e | Infant Formula | |  | f | Tea/infusion/”hamesa” | |  | g | Fresh butter | |  | h | Other | |
| Specify other  Question relevant when: regex( ${wq822} ,'^.*h.*$') |  |
| q823. Did you do something to keep [q104_w0_11] warm following delivery? | |  | 1 | Yes 2. No | | --- | --- | --- | |  | 98 | Don’t know | |
| q823. What did you do to keep the baby [q104_w0_11] warm following delivery?  Do not read responses; ASK: Anything else?; Record all responses  Question relevant when: ${wq823a} =1 | |  | a | Dried the baby | | --- | --- | --- | |  | b | Wrapped the baby with clean cloth | |  | c | Put baby beside the mother | |  | d | Keep the baby on bare skin to skin contact | |  | e | Bathed in warm water | |  | f | Warmed delivery room | |  | g | Other | |
| q824. In the first fifteen days of life how frequently per day did you hold [q104_w0_11] skin-to-skin against breast during the daytime and nighttime? | |  | 1 | Always | | --- | --- | --- | |  | 2 | Often | |  | 3 | A few times | |  | 4 | Never | |  | 5 | Don’t know | |
| q825. In the first fifteen days did you sleep with [q104_w0_11] against you at night, or did lay him/her alone on the bed, or elsewhere?  Mainly with whom did he sleep | |  | 1 | Mother slept with baby | | --- | --- | --- | |  | 2 | Laid baby on bed alone | |  | 3 | Baby slept with another person | |  | 4 | Keep the baby on bare skin to skin contact | |  | 5 | Other (specify) | |
| q826. What do you think are the complications in a woman during childbirth needing medical treatment?  Do not read responses; ASK: Anything else?; Record all responses | |  | a | Excessive vaginal bleeding | | --- | --- | --- | |  | b | Foul-smelling discharge | |  | c | High fever | |  | d | Baby’s hand or feet come first | |  | e | Baby’s in abnormal position | |  | f | Prolonged labor (>12 hours) | |  | g | Retained placenta | |  | h | Rupture uterus | |  | i | Prolapsed cord | |  | j | Cord around neck | |  | l | Convulsions | |  | m | loss of consciousness | |  | n | Preterm | |  | o | Preterm premature rapture of memberane (pPROM) | |  | p | Nothing mentioned | |  | k | Other (specify) | |
| q827. Do you know where to go if you experienced delivery complication, e.g. sever head ache, sever vaginal bleeding, prolonged labor or retained placenta your? | |  | 1 | Yes | | --- | --- | --- | |  | 2 | No | |
| q827. Where can you go if you experienced delivery complication, e.g. sever head ache, sever vaginal bleeding, prolonged labor or retained placenta your?  Do not read responses; ASK: Anything else?; Record all responses  Question relevant when: ${wq827a} =1 | |  | a | Hospital | | --- | --- | --- | |  | b | Health center | |  | c | Health post | |  | d | Drug shop/pharmacy | |  | f | Health development army leaders (HDAs) | |  | g | TBA/TTBA | |  | h | Holy water/religious place | |  | i | Traditional healer/Witchcraft | |  | j | Other(specify) | |
| Did you have any of the symptoms of the danger signs during the birth of [q104_w0_11]? | |  | 1 | Yes 2. No | | --- | --- | --- | |  | 8 | Don’t know | |
| q828. Which of the following symptoms you faced during delivery of [q104_w0_11]?  -  Question relevant when: ${wq828cbexp} =1 | |  | a | Excessive vaginal bleeding | | --- | --- | --- | |  | b | Foul-smelling discharge | |  | c | High fever | |  | d | Baby’s hand or feet come first | |  | e | Baby’s in abnormal position | |  | f | Prolonged labor (>12 hours) | |  | g | Retained placenta | |  | h | Rupture uterus | |  | i | Prolapsed cord | |  | j | Cord around neck | |  | l | Convulsions | |  | m | loss of consciousness | |  | n | Preterm | |  | o | Preterm premature rapture of memberane (pPROM) | |  | p | Nothing mentioned | |  | k | Other (specify) | |
| wq829a. When you had the symptoms, did you go some where to seek care?  Question relevant when: ${wq828cbexp} =1 | |  | 1 | Yes | | --- | --- | --- | |  | 2 | No | |
| q829. Where did you go when you had any of the symptoms?  Question relevant when: ${wq828cbexp} =1 and ${wq829a} =1 | |  | a | Hospital | | --- | --- | --- | |  | b | Health center | |  | c | Health post | |  | d | Drug shop/pharmacy | |  | f | Health development army leaders (HDAs) | |  | g | TBA/TTBA | |  | h | Holy water/religious place | |  | i | Traditional healer/Witchcraft | |  | j | Other(specify) | |
| Postnatal care for mother |  |
| q900. Did you or anyone from your family or neighborhood inform the HEW about your child birth immediately after delivery?  If the delivery is home deliver  Question relevant when: ${wq801} =1 or ${wq801} =2 | |  | 1 | Yes 2. No | | --- | --- | --- | |  | 8 | Don’t know | |
| q901. Did you or anyone from your family or neighborhood inform the HEW about your child birth immediately after delivery?  For Facility delivery  Question relevant when: ${wq801} =3 or ${wq801} =4 or ${wq801} =5 or ${wq801} =6 or ${wq801} =7 or ${wq801} =8 or ${wq801} =9 | |  | 1 | Yes 2. No | | --- | --- | --- | |  | 8 | Don’t know | |
| For how long did you stay in the facility after the delivery?  In Hours. Enter 72 if >72  Question relevant when: ${wq801} =3 or ${wq801} =4 or ${wq801} =5 or ${wq801} =6 or ${wq801} =7 or ${wq801} =8 or ${wq801} =9  Response constrained to: .<73 |  |
| Did anyone check the baby's health while you were still in the facility  -  Question relevant when: ${wq801} =3 or ${wq801} =4 or ${wq801} =5 or ${wq801} =6 or ${wq801} =7 or ${wq801} =8 or ${wq801} =9 | |  | 1 | Yes 2. No | | --- | --- | --- | |  | 8 | Don’t know | |
| Did any health care provider check on your own health within 6 weeks of giving birth?  - | |  | 1 | Yes | | --- | --- | --- | |  | 2 | No | |
| How many days after giving birth did you have your first health check?  Enter number of days , Enter 99 if not known  Question relevant when: ${wq904} =1  Response constrained to: .<43 or .=99 |  |
| Where did the first check take place?  Question relevant when: ${wq904} =1 | |  | 1 | In own home | | --- | --- | --- | |  | 2 | Health post | |  | 3 | Health center | |  | 4 | Hospital | |  | 5 | Other | |
| By whom?  Question relevant when: ${wq904} =1 | |  | 1 | Doctor | | --- | --- | --- | |  | 2 | Nurse/midwife | |  | 3 | HEW | |  | 4 | Health Officer | |  | 5 | HDA | |  | 8 | TBA | |  | 6 | Do not know | |  | 7 | Other | |
| Specify other  Question relevant when: ${wq907} =7 |  |
| Was a second visit conducted?  -  Question relevant when: ${wq904} =1 | |  | 1 | Yes | | --- | --- | --- | |  | 2 | No | |
| How many days after giving birth did you have your second health check?  Enter number of days , Enter 99 if not known  Question relevant when: ${wq904} =1 and ${wq908} =1  Response constrained to: .<43 or .=99 and .> ${wq905} or .= ${wq905} |  |
| Where did the checking take place?  Question relevant when: ${wq904} =1 and ${wq908} =1 | |  | 1 | In own home | | --- | --- | --- | |  | 2 | Health post | |  | 3 | Health center | |  | 4 | Hospital | |  | 5 | Other | |
| By whom?  Question relevant when: ${wq904} =1 and ${wq908} =1 | |  | 1 | Doctor | | --- | --- | --- | |  | 2 | Nurse/midwife | |  | 3 | HEW | |  | 4 | Health Officer | |  | 5 | HDA | |  | 8 | TBA | |  | 6 | Do not know | |  | 7 | Other | |
| Was a third visit conducted?  -  Question relevant when: ${wq904} =1 and ${wq908} =1 | |  | 1 | Yes | | --- | --- | --- | |  | 2 | No | |
| How many days after giving birth did you have your third health check?  Enter number of days , Enter 99 if not known  Question relevant when: ${wq904} =1 and ${wq908} =1 and ${wq912} =1  Response constrained to: .<43 or .=99 and .> ${wq909} or .= ${wq909} |  |
| Where did the checking take place?  Question relevant when: ${wq904} =1 and ${wq908} =1 and ${wq912} =1 | |  | 1 | In own home | | --- | --- | --- | |  | 2 | Health post | |  | 3 | Health center | |  | 4 | Hospital | |  | 5 | Other | |
| Specify other  Question relevant when: ${wq914} =5 |  |
| By whom?  -  Question relevant when: ${wq904} =1 and ${wq908} =1 and ${wq912} =1 | |  | 1 | Doctor | | --- | --- | --- | |  | 2 | Nurse/midwife | |  | 3 | HEW | |  | 4 | Health Officer | |  | 8 | TBA | |  | 6 | Do not know | |  | 7 | Other | |
| During visits after giving birth, what was done to check your health?  Read List out loud and Mark all that apply  Question relevant when: ${wq904} =1 | |  | a | Checked breasts | | --- | --- | --- | |  | b | Advised breast feeding | |  | c | Oriented about danger signs | |  | d | Educated on family planning | |  | e | Information given on nutrition | |  | f | Referred to a health facility | |  | g | Measured Blood Pressure | |  | h | Checked/treated birth-related wound (if applicable) | |  | j | Family planning counselling | |  | k | Assessed temperature | |  | l | Assessed breathing | |  | i | Other | |
| specify other  Question relevant when: regex( ${wq916} ,'^.*i.*$') |  |
| Can you tell us your satisfaction or dissatisfaction for the postnatal care you received?  Question relevant when: ${wq904} =1 | |  | 1 | Fully satisfied | | --- | --- | --- | |  | 2 | Somewhat satisfied | |  | 3 | Somewhat dissatisfied | |  | 4 | Fully dissatisfied | |
| In the first 6 weeks after [q104_w0_11] was born, did any health care provider excluding traditional birth attendant check on [q104_w0_11] health?  - | |  | 1 | Yes | | --- | --- | --- | |  | 2 | No | |
| If YES, how many days after birth was [q104_w0_11] 's first health check?  Enter number of days and Enter 99 if not known  Question relevant when: ${wq918} =1  Response constrained to: .<43 or .=99 |  |
| If YES, where did the health checks on [q104_w0_11] take place?  Question relevant when: ${wq918} =1 | |  | 1 | In own home | | --- | --- | --- | |  | 2 | Health post | |  | 3 | Health center | |  | 4 | Hospital | |  | 5 | Other | |
| If YES, who checked on the health of [q104_w0_11] ?  -  Question relevant when: ${wq918} =1 | |  | 1 | Doctor | | --- | --- | --- | |  | 2 | Nurse/midwife | |  | 3 | HEW | |  | 4 | Health Officer | |  | 5 | HDA | |  | 8 | TBA | |  | 6 | Do not know | |  | 7 | Other | |
| Was a second check conducted?  Question relevant when: ${wq918} =1 | |  | 1 | Yes | | --- | --- | --- | |  | 2 | No | |
| If YES, how many days after birth was [q104_w0_11] 's second health check?  Enter number of days , Enter 99 if not known  Question relevant when: ${wq918} =1 and ${wq922} =1  Response constrained to: .> ${wq919} or .= ${wq919} and .<43 or .=99 |  |
| If YES, Where did the health checks on [q104_w0_11] take place?  Question relevant when: ${wq918} =1 and ${wq922} =1 | |  | 1 | In own home | | --- | --- | --- | |  | 2 | Health post | |  | 3 | Health center | |  | 4 | Hospital | |  | 5 | Other | |
| If YES, Who checked on the health of [q104_w0_11]?  Question relevant when: ${wq918} =1 and ${wq922} =1 | |  | 1 | Doctor | | --- | --- | --- | |  | 2 | Nurse/midwife | |  | 3 | HEW | |  | 4 | Health Officer | |  | 5 | HDA | |  | 8 | TBA | |  | 6 | Do not know | |  | 7 | Other | |
| Was a third check conducted?  Question relevant when: ${wq918} =1 and ${wq922} =1 | |  | 1 | Yes | | --- | --- | --- | |  | 2 | No | |
| If YES- how many days after birth was [q104_w0_11] 's third health check?  Enter number of days , Enter 99 if not known  Question relevant when: ${wq918} =1 and ${wq926} =1  Response constrained to: .> ${wq923} or .= ${wq923} and .<43 or .=99 |  |
| If YES, Where did the health checks on [q104_w0_11] take place?  Question relevant when: ${wq918} =1 and ${wq926} =1 | |  | 1 | In own home | | --- | --- | --- | |  | 2 | Health post | |  | 3 | Health center | |  | 4 | Hospital | |  | 5 | Other | |
| If YES, Who checked on the health of [q104_w0_11]?  -  Question relevant when: ${wq918} =1 and ${wq926} =1 | |  | 1 | Doctor | | --- | --- | --- | |  | 2 | Nurse/midwife | |  | 3 | HEW | |  | 4 | Health Officer | |  | 5 | HDA | |  | 8 | TBA | |  | 6 | Do not know | |  | 7 | Other | |
| At any of the health checks, what was done to check the health of [q104_w0_11]?  Read List out loud and Mark all that apply  Question relevant when: ${wq918} =1 | |  | a | Generally examined /looked at baby's body | | --- | --- | --- | |  | b | Weighed baby | |  | c | Checked cord | |  | d | Advised breastfeeding/exclusive breast feeding | |  | e | Observed breastfeeding | |  | f | Advised skin-to-skin contact/warmth | |  | g | Checked baby for danger signs (including sepsis) | |  | h | Educated on danger signs | |  | i | Referred to a health facility | |  | j | Provided information on washing hands before touching baby | |  | k | Advised keeping the cord clean | |  | l | Advised not to bathe the baby within 24 hours after birth | |  | n | Child immunization | |  | m | other | |
| Can you tell us your satisfaction or dissatisfaction for the postnatal care given for your baby?  Question relevant when: ${wq918} =1 | |  | 1 | Fully satisfied | | --- | --- | --- | |  | 2 | Somewhat satisfied | |  | 3 | Somewhat dissatisfied | |  | 4 | Fully dissatisfied | |
| The last time you gave birth, did you keep your newborn at home for several days or weeks without taking the baby out?  - | |  | 1 | Yes | | --- | --- | --- | |  | 2 | No | |
| What is the number of days that you kept your newborn in the house?  Ask all respondents  Response constrained to: .<45 |  |
| The last time you gave birth, were there visitors come to your house to see the baby? This includes visitors for any reason: health care workers, extended family, or friends.  - | |  | 1 | Yes | | --- | --- | --- | |  | 2 | No | |
| The last time you gave birth, how many days passed before you had visitors come to your house to see the baby? This includes visitors for any reason: health care workers, extended family, or friends.  Enter '0' if lessthan a day and 99 if do not know  Question relevant when: ${wq934vist} =1  Response constrained to: .>=0 and .<=42 or .=99 |  |
| The last time you gave birth, someone other than you had physical contact with the baby?  - | |  | 1 | Yes | | --- | --- | --- | |  | 2 | No | |
| The last time you gave birth, how many days passed before someone other than you had physical contact with the baby?  Enter 99 if do not know  Question relevant when: ${wq935tou} =1  Response constrained to: .>=0 and .<=42 or .=99 |  |
| When did the health worker/HDA discuss with about family planning method that can be taken after giving birth? | |  | 1 | Within 24 hours after delivery | | --- | --- | --- | |  | 2 | within 3 days after delivery | |  | 3 | within 1 week after delivery | |  | 4 | within 6 weeks after delivery | |  | 5 | beyond 6 weeks after delivery | |  | 6 | Not discussed on family planning | |
| About which post partum family planning methods the health worker/HEWs discuss with you?  Question relevant when: ${wq620} =1 | |  | 1 | Female Sterilization | | --- | --- | --- | |  | 2 | Male Sterilization | |  | 3 | Pill | |  | 4 | IUD | |  | 5 | Injections | |  | 6 | Implants | |  | 7 | Condom | |  | 8 | Diaphragm/Foam/Jelly | |  | 9 | Periodic Abstinence | |  | 10 | Withdrawal | |  | 11 | LAM | |  | 13 | Do not know | |  | 14 | Emergency contraception | |  | 15 | Standard days method/Cycle Beads | |  | 12 | Other | |
| Now I am going to ask you on the newborn health care seeking issues |  |
| If a newborn infant is healthy, there is no need to take the newborn to the health center (or health post). | |  | 1 | Agree a lot | | --- | --- | --- | |  | 2 | Agree somewhat | |  | 3 | Disagree somewhat | |  | 4 | Disagree a lot | |
| A newborn infant should not be taken to the health center or health post for some days for cultural or religious reason or belief?  Mention example like waiting till baptism or any other cultural or religious reason. | |  | 1 | Agree a lot | | --- | --- | --- | |  | 2 | Agree somewhat | |  | 3 | Disagree somewhat | |  | 4 | Disagree a lot | |
| wq940. Sometimes mothers after delivery have severe illnesses and should be taken immediately to a health facility. What type of symptoms would cause mothers to go to a health facility right away?  Do not read responses; ASK: Anything else?; Record all responses | |  | a | Excessive vaginal bleeding | | --- | --- | --- | |  | b | Foul-smelling discharge | |  | c | High fever | |  | d | Severe lower abdominal pain | |  | e | Convulsions | |  | h | Unconsciousness | |  | i | Severe headache | |  | j | Blurred vision | |  | k | Calf pain | |  | l | postpartum psychosis | |  | g | Nothing mentioned | |  | f | Other | |
| wq940a. Did you face illness after delivery that needs immediate medical attention? | |  | 1 | Yes | | --- | --- | --- | |  | 2 | No | |
| wq940b. Which of the following symptoms did you face?  Question relevant when: ${wq940apnc} =1 | |  | a | Excessive vaginal bleeding | | --- | --- | --- | |  | b | Foul-smelling discharge | |  | c | High fever | |  | d | Severe lower abdominal pain | |  | e | Convulsions | |  | h | Unconsciousness | |  | i | Severe headache | |  | j | Blurred vision | |  | k | Calf pain | |  | l | postpartum psychosis | |  | g | Nothing mentioned | |
